# Improved X-ray baggage screening sensitivity with ‘targetless’ search training

**DOI:** 10.1186/s41235-021-00295-0

**Published:** 2021-04-14

**Authors:** Alex Muhl-Richardson, Maximilian G. Parker, Sergio A. Recio, Maria Tortosa-Molina, Jennifer L. Daffron, Greg J. Davis

**Affiliations:** Department of Psychology, University of Cambridge, Downing Street, Cambridge, CB2 3EB UK

**Keywords:** Targetless search, X-ray baggage search, Visual search, Target templates, Templates for rejection, Distractor templates

## Abstract

When searching for a known target, mental representations of target features, or templates, guide attention towards matching objects and facilitate recognition. When only distractor features are known, distractor templates allow irrelevant objects to be recognised and attention to be shifted away. This is particularly true in X-ray baggage search, a challenging real-world visual search task with implications for public safety, where targets may be unknown, difficult to predict and concealed by an adversary, but distractors are typically benign and easier to identify. In the present study, we draw on basic principles of distractor suppression and rejection to investigate a counterintuitive ‘targetless’ approach to training baggage search. In a simulated X-ray baggage search task, we observed significant benefits to target detection sensitivity (*d′*) for targetless relative to target-based training, but no effects of performance-contingent rewards or the inclusion of superordinate semantic categories during training. The benefits of targetless search training were most apparent for stimuli involving less spatial overlap (occlusion), which likely represents the difficulty and greater individual variation involved in searching more visually complex images. Together, these results demonstrate the effectiveness of a counterintuitive targetless approach to training target detection in X-ray baggage search, based on basic principles of distractor suppression and rejection, with potential for use as a real-world training tool.

**Supplementary Information:**

The online version contains supplementary material available at 10.1186/s41235-021-00295-0.

X-ray baggage search, the process of visually searching X-ray images of baggage for threats and prohibited items, is an important component of airport security that helps to ensure the safety of aircraft and passengers. Compared to other real-world visual search tasks, such as medical screening, baggage search involves some unique challenges, both due to how the task is specified and the nature of the visual environment.

One of the challenges in baggage search arises due to how the task is typically specified, i.e. in terms of a wide range of visually diverse and somewhat unpredictable targets that must be detected. There is an extensive literature on dual-target (and multiple-target) costs in visual search in both basic and applied tasks (Barrett & Zobay, 2014; Godwin et al., 2010; Menneer et al., 2007, 2009; Stroud et al., 2012), and such a cost is inevitably involved in baggage search as screeners are required to detect the presence of any target from a long list of prohibited items. However, comparatively high variation within some target categories may mean that some items within these categories are more difficult to detect (Hout & Goldinger, 2015) and, while some target categories can be quite precisely specified due to high within-category *homogeneity*, for many, high within-category *heterogeneity* is much more likely.

Developing broad categorical target templates that represent features common to a category is difficult for heterogenous categories. For example, pistols represent a comparatively homogenous target category and their features are easily predictable (mostly metal, small range of possible shapes). On the other hand, improvised explosives are much more heterogenous and their features can be difficult to predict. They may take any one of an almost unlimited number of forms and may share almost no features with other improvised explosives. Developing a categorical template that will support guidance towards and recognition of this type of target is difficult (Hout et al., 2017) and may limit the effectiveness of target-based training procedures.

The literature on distractor suppression in basic search tasks shows that, independently of target templates, searchers can use knowledge of distractors to build distractor templates (also called templates for rejection) to guide attention away from distractors (Arita et al., 2012; Daffron & Davis, 2015; Geng, 2014; Moher & Egeth, 2012). This voluntary process is distinct from the suppression of bottom-up attentional capture by irrelevant salient stimuli that has been the subject of recent work in this area (Chang & Egeth, 2019; Gaspelin et al., 2015). Sometimes this might involve initial attention towards and recognition of distractors, such that they might be rejected reactively, sometimes called a ‘search and destroy’ strategy, but alternatively, distractor rejection might be more proactive, with early attention towards distractors suppressed so that targets (known or unknown) might be located and recognised more readily. Regardless of the mechanism, distractor templates appear to facilitate the detection of novel search targets and can operate *in the absence of a functional target template*, which may be incredibly useful in tasks where it is difficult or impossible to specify or learn target features. Implicit forms of distractor suppression, for example, visual marking (Watson & Humphreys, 1997, 2000) and distractor-previewing effects (Ariga & Kawahara, 2004; Goolsby & Suzuki, 2001, 2002) may contribute to the development of distractor templates in addition to more explicit processes.

The present study focusses on distractor templates, which may be particularly important in baggage search for two reasons. Firstly, screeners almost exclusively encounter non-targets, as the vast majority of bags do not contain threats, and therefore experience a continuous steam of opportunities to learn about non-target features that can inform and support categorical distractor templates. Screeners might not experience a genuine target in their entire career, with simulated targets providing the only on-task learning opportunities. Secondly, the detection of novel or unusual targets (which often cannot be well specified in advance) would not rely on knowledge of target features, but rather the features of the much more familiar non-targets amongst which they appear.

Previous studies of distractor templates have involved basic laboratory search tasks, but it is difficult to predict how effective they may be in baggage search due to the unique combination of challenges to the human observer posed by this task. Perhaps the most fundamental of these is that baggage X-rays lack a regular structure (Donnelly et al., 2019). In laboratory tasks, everyday searches and medical imaging, the search environment involves some predictable structure, e.g. a radiographer examining a lung CT (computed tomography) scan knows how a human lung is typically arranged; in comparison, baggage screeners may have very few valid expectations about where items might appear within an image (McCarley et al., 2004).

Baggage X-rays typically follow a standard colour mapping, whereby objects are coloured based upon their density and absorption of X-ray radiation (Donnelly et al., 2019). By this mapping, metallic objects (higher density) are coloured blue and organic objects (lower density) are orange. Some items of moderate density are coloured green and extremely high-density objects appear as black. This colour mapping creates a novel search environment in which object appearance can differ significantly from expectations based on the normal visual world. Furthermore, all except extremely dense items have some degree of transparency, meaning that the appearance of almost every item will be influenced by spatially overlapping items, including the bag or container. Not only can overlap (occlusion) make it more difficult to segment images and identify object boundaries, but it also influences the colour of overlapping objects, which is typically based on averages of the relevant item properties (Godwin et al., 2017). These complexities and the lack of reliable cues that might be available in other visual scenes mean that search guidance in baggage X-rays is likely extremely limited (Vickery et al., 2005; J. Wolfe et al., 2008).

There is a large body of work on effects related to the extremely low prevalence of threats in baggage (Fleck & Mitroff, 2007; Godwin et al., 2010, 2015; Menneer et al., 2010; Mitroff & Biggs, 2014; Wolfe et al., 2007, 2013). In visual search tasks, low levels of target prevalence (i.e. when targets are rare) are associated with reduced hit rates (and false alarm rates) due to a conservative shift in response criterion. Eye movement studies went on to reveal that this criterion shift is associated with errors of perceptual selection, whereby targets are less likely to be fixated before the task is terminated, and of perceptual identification, whereby targets are less likely to be correctly identified following fixation (Godwin et al., 2015). While the effect of low prevalence in real-world baggage screening may be ameliorated through procedures that artificially increase threat prevalence (e.g. Threat Image Projection), it nonetheless remains a challenge for screeners (Donnelly et al., 2019). A related literature exists on satisfaction of search effects (also referred to as subsequent search misses; Adamo et al., 2018; Cain et al., 2013; Fleck et al., 2010), whereby searchers are less likely to detect subsequent targets after finding an initial target. However, real-world baggage search remains principally concerned with finding any single initial target, as this will always be sufficient to identify dangerous baggage.

While previous studies have examined X-ray baggage search from a human factors perspective (e.g. Buser et al., 2020; Hättenschwiler et al., 2019; Schwaninger, 2016), the present study takes a psychological approach by investigating the potential benefits of distractor templates in this context. We develop and test a targetless search training procedure for novice screeners, focussed on making use of broad categorical distractor templates in simulated X-ray baggage search tasks, incorporating some of the challenges discussed above. Acknowledging the severe limitations imposed on guidance when searching baggage X-rays, the focus of our targetless training procedure is not improving search guidance, but training the recognition (or identification) step of the search process, specifically with a focus on improving recognition of safe items rather than threats.

The current experiments build on unpublished pilot findings using the same rationale but with photographic stimuli, suggesting that a consequence of training distractor recognition is improved detection of challenging targets. The final experiment attempts to incorporate the preceding results to develop and test an enhanced targetless search training procedure. We examine performance both in terms of behavioural responses and Signal Detection Theory measures of sensitivity (*d′*) and bias (c).

## Experiment 1

Experiment 1 aimed to test whether participants could be trained to make use of distractor templates, improving distractor recognition and, critically, aiding the detection of target objects (threat items that are prohibited in cabin baggage) in a simulated baggage search task. To do this, we developed a targetless search training procedure that focussed on training novice participants to recognise non-targets (safe items that are permitted in cabin baggage). We compared this targetless search training procedure with target-based search training (focussed exclusively on target recognition) and combined search training (including elements of target and non-target recognition). In order to avoid pre-existing biases for search strategies involving search *for* targets rather than rejecting non-targets and given a lack of baggage screening experience in our sample, we did not use a pre-/post-training test design and instead tested participants once following training. We expected that if participants naturally adopted a strategy based on searching for targets in a pre-training test, then this would potentially reduce the effectiveness of search training focussed on the rejection of non-targets.

We predicted that, relative to target-based search training, participants who received targetless search training would use distractor templates to recognise and exclude non-targets and therefore be better able to detect the presence of novel (untrained) targets in the simulated baggage search task. We also predicted that participants who received the combined search training would perform more similarly to those who received target-based training than targetless training due to a bias in favour of the target-based approach to search, i.e. searching *for* a target. Finally, we predicted that a conventional prevalence effect would be observed, such that low prevalence target categories would be associated with a lower hit rate than high prevalence target categories.

### Method

#### Participants

Sixty participants (42 females, 18 males; *M*_age_ = 22.98 years, SD = 5.23) were recruited and randomly allocated to one of three training groups of equal size. Participants were recruited via the Department of Psychology Research Sign-up System and were reimbursed £10 for their time. All experiments presented in this manuscript were approved by the Ministry of Defence Research Ethics Committee and the Cambridge Psychology Research Ethics Committee.

#### Apparatus and stimuli

The experiment was programmed using PsychoPy 1.90.3 (Peirce, 2007, 2009) and presented on a 24″ Dell LCD monitor. Participants viewed the monitor from a distance of approximately 70 cm and responded using a keyboard.

The stimuli used for the familiarisation phase were taken from the CaSePIX X-ray image library, which we created using a Todd Research TR70 conveyor X-ray machine. These familiarisation phase stimuli consisted of a subset of 11 X-ray images of empty suitcases, with features such as zips and wheels labelled.

Further stimuli were created using SimFox (Renful Premier Technologies), web-based software used for real-world screener training. SimFox included an X-ray image library of baggage and items that can appear in baggage (including a range of threat and safe items) and we also used it to generate realistic composite images of baggage containing multiple items. Bag stimuli ranged in size from approximately 5.5° to 15.5° of visual angle in both dimensions and individual objects presented alone ranged in size from 0.5° to 16.8° degrees of visual angle in both dimensions.

We began by defining two sets of object categories, 14 threat categories and 14 safe categories (see Additional file 1: Supplementary Tables S1–S5). The training phase stimuli were X-ray images of individual objects that fitted into these categories and in total consisted of 196 threat objects and 196 safe items (participants only ever viewed a subset of these stimuli that was dependent upon their training group, see Design and Procedure). The testing phase stimuli were composite X-ray images of bags and were made to contain six individual objects. In total 168 testing phase bags were generated, 84 containing a single object from one of the threat categories (plus five safe items) and 84 containing only safe items (i.e. 50% overall threat prevalence).

Threat objects that were used in the testing phase bags were not used in the training phase and were not repeated between testing phase bags. Safe items could appear in up to five different testing phase bags and some safe items in testing phase bags also appeared during training. We reserved some safe items for the test phase only and, for other items, limited the number of appearances in the training phase. Across all bag stimuli presented at test, this resulted in: 21 bags with entirely novel (unseen during training) safe items, 53 bags with only one safe item presented during training (five items entirely novel), 58 bags with two safe items presented during training, 27 bags with three safe items presented during training, seven bags with four safe items presented during training and two bags with five safe items presented during training.

While this approach treated threat and safe items differently across training and test phases, it not only allowed us to generate a large number of stimuli, but also meant that these stimuli that better represented real-world baggage screening conditions, where categories of safe item are often highly homogenous (compared to threat items), some of the same safe items may be found in many different bags (e.g. popular brands of mobile phone or laptop computer) and specific threats are difficult to predict or foresee.

While the overall prevalence of threat objects was held at 50%, the relative prevalence of specific categories of threat was manipulated. In all test phases for Experiments 1 to 4, there were seven high prevalence threat categories (brackets show percentage of trials with target present rounded to nearest integer): explosives (5%), firearm magazines/components (6%), large firearms (3%), small firearms (7%), knives/stabbing weapons (7%), liquids/gases (4%), snips/scissors/pliers (5%). There were also seven low prevalence threat categories: grenades (2%), ammunition (2%), blunt weapons/axes (2%), throwing stars/knuckles (2%), power tools (2%), shrapnel (2%), trowels/wrenches (2%). For all experiments, high prevalence threat categories at test also had high prevalence during training and low prevalence threat categories at test also had low prevalence at training, although precise prevalence levels varied due to the number of trials and the number of available stimuli in each category (see Additional file 1: Supplementary Tables S1–S5 for exact numbers of stimuli in each category in all phases/experiments).

#### Design and procedure

**Fig. 1:**
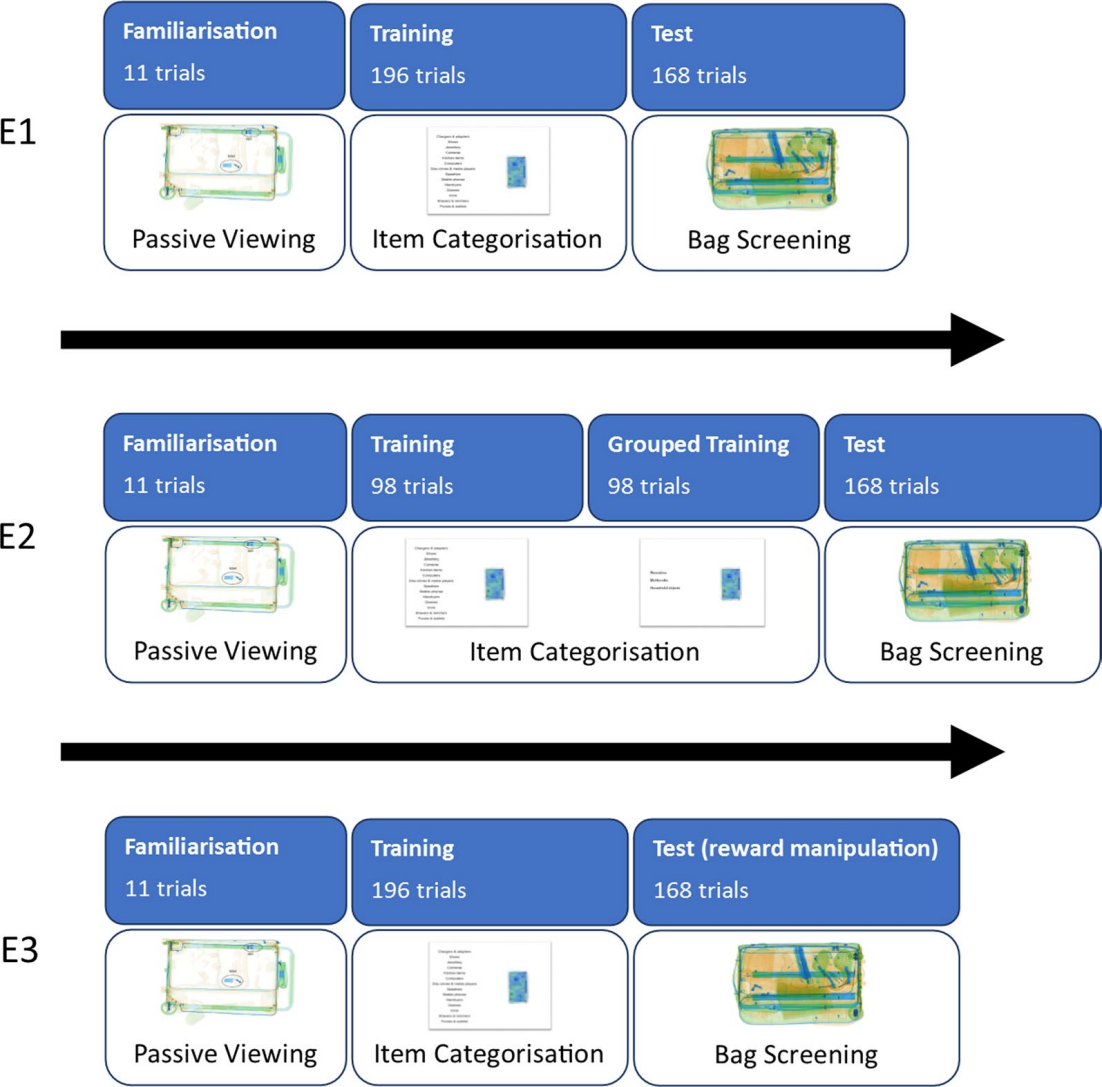
Outline Procedures for Experiments 1 to 3 (see Fig. 2 for a more detailed example of an item categorisation trial)

To minimise the likelihood of participants autonomously adopting a standard target-based approach to search prior to training (that could potentially persist after training), Experiment 1 did not utilise a pre-training test phase (see Fig. 1, figure includes stimuli not used in the experiment shown for illustrative purposes only). Search performance was instead assessed after training with a single test phase. Participants were randomly assigned to one of three training groups, one of which included only threat items (target-based search training), one of which included only safe items (standard targetless search training; sTST) and one of which included a combination of threat and safe items (combined search training; CST).

**Fig. 2:**
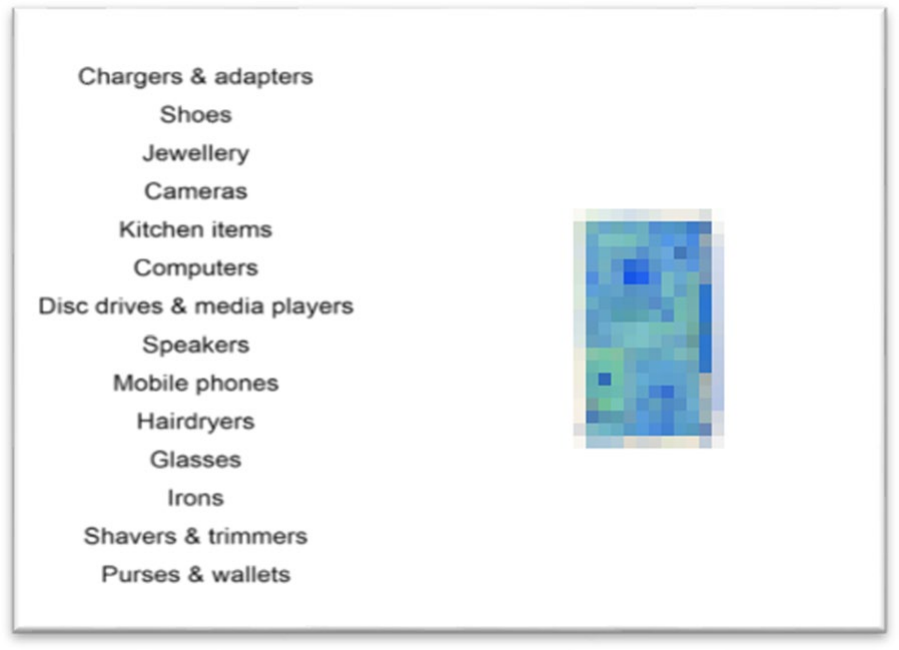
Example item categorisation training trial from Experiment 1 (also used in Experiments 2, 3 and 4), participants were required to click on the category which matched the object (pixelised stimulus shown for illustrative purposes only—we are unable to share the stimuli used here due to the terms of our license with a commercial provider)

Participants initially completed a short familiarisation phase which involved passively viewing 11 X-ray images of empty suitcases (with some features labelled). These were viewed sequentially and viewing was self-paced. The rationale behind this phase was to familiarise participants both with the appearance of baggage X-ray images in general and with some of the specific features of suitcases, which form the ‘background’ of all stimuli used in the test phase.

Following completion of the familiarisation phase, participants began the training phase. On each trial of the training phase, a single X-ray image of an object (see Apparatus and Stimuli) was presented against a white background and participants were required to indicate the category to which the object belonged by using the mouse to click on one of 14 category labels presented in a list on the left of the screen (see Fig. 2; the category labels present depended on the training group and block). Following the click, feedback was provided which either stated that the selection was correct or indicated what the correct category was if the response was incorrect. The target-based search group completed 196 training trials categorising threat items, the sTST group completed 196 training trials categorising safe items and the CST group completed 98 training trials categorising threat items and 98 training trials categorising safe items. A single object was displayed on each trial and each object was displayed only once. These trials were organised into four equal sized blocks and for the CST group, threat and safe items were presented in separate blocks that were order counterbalanced. The target-based search group did not learn about the 14 safe item categories and the sTST group did not learn about the 14 threat object categories, but the CST group did learn about all 28 object categories.

Following training, participants completed a test phase to assess their performance. All participants were informed that the test phase would involve viewing bags and determining whether they were ‘safe’ or ‘dangerous’ and that bags should be considered safe if they contained only items that were typically allowed in aircraft cabin baggage and dangerous if they contained an item that was typically prohibited in this situation. All participants were instructed to use what they had learned in the training phase to help them complete the test phase, specifically, the target-based search group was instructed to focus on identifying the dangerous items they learned about and to treat items they did not recognise as safe, the sTST group was instructed to focus on ignoring the safe items they learned about and to treat items they did not recognise as dangerous, and the CST group was instructed to focus on identifying the dangerous items and ignoring the safe items they learned about. No instructions were given about threat or safe item prevalence or frequency. In each test trial of the test phase, a central fixation cross was presented for one second, followed by an X-ray image of a bag containing six items (see Apparatus and Stimuli) presented for five seconds. After this time the bag stimulus disappeared, and the participant was prompted to respond to indicate whether the bag was ‘dangerous’ or ‘safe’ by pressing either the ‘z’ or ‘m’ key on the keyboard. Following the experiment, participants were debriefed, informed of the aims of the study and given the opportunity to ask any questions. As a whole the experiment lasted no more than one hour per participant.

### Results

We first tested whether the benefits of sTST might reflect repetition of items from that group’s training. To do this, we identified the 74 test phase bags which included zero or one safe items presented during sTST training (‘low/no repeat’ bags) and a further 94 bags which included two or more safe items presented during sTST training (‘high repeat’ bags). Any benefit for the sTST group that derived from benefit of training items should be most evident in the high repeat set of bags. We calculated *d′* separately for these two sets of test stimuli, across sTST and target-based training groups (the latter of which had not viewed any safe items prior to test), and conducted a two-way mixed ANOVA with training group (sTST, target-based) and item repetition (high repeat, low/no repeat) as factors. This yielded main effects of training group, *F*(1,38) = 22.03, *p* < 0.001, *η*_*G*_^2^ = 0.31, and of item repetition, *F*(1,38) = 28.60, *p* < 0.001, *η*_*G*_^2^ = 0.15. However, there was no interaction between training group and stimulus repetition, *F*(1,38) = 2.06, *p* = 0.159. This provided no evidence that high repeat bags (*M*_sTST_ = 1.55, SD_sTST_ = 0.25, *M*_target-based_ = 1.17, SD_target-based_ = 0.22) conferred a specific advantage for the sTST group over the target-based group relative to low/no repeat bags (*M*_sTST_ = 1.34, SD_sTST_ = 0.25, *M*_target-based_ = 0.81, SD_target-based_ = 0.22). While item repetition did have a main effect on *d′* this was consistent across training groups, despite all safe items being novel at test for the target-based group. This likely reflects a combination of stimulus specific effects and recognition benefits accumulated during the test phase for objects which appeared in multiple test phase bags. In any case, stimulus repetition does not appear to explain the benefits conferred by sTST. Further analysis of these differences for bags that contained no repeated items and only a single repeated item is included in Additional file 1: Table S5.

Analysis of sensitivity (*d′*) and criterion (*c*) revealed higher *d′* scores for those who received sTST and CST, relative to target-based training, and criterion differences between the training groups, mostly notably between the target-based training and sTST groups which were biased towards responding that bags contained the types of item they had viewed during training.

We conducted two between-subjects ANOVAs. There was a significant effect of training group on *d′*, *F*(2,57) = 12.99, *p* < 0.001, *η*_*G*_^2^ = 0.31. Planned comparisons revealed that *d′* for the target-based search training group (*M* = 1.00, SD = 0.25) was significantly lower than both the sTST group (*M* = 1.46, SD = 0.38), *t*(38) = 4.56, *p* < 0.001, *d* = 1.44, and the CST group (*M* = 1.45, SD = 0.33), *t*(38) = 4.77, *p* < 0.001, *d* = 1.51. There was no significant difference between the sTST group and the CST group, *t*(38) = 0.17, *p* = 0.867.

There was a significant effect of training group on *c*, *F*(2,57) = 17.95, *p* < 0.001, *η*_*G*_^2^ = 0.39. Planned comparisons revealed that *c* was significantly lower for the target-based search training group (*M* =  − 0.18, SD = 0.39) than both the sTST group (*M* = 0.42, SD = 0.32), *t*(38) = 5.23, *p* < 0.001, *d* = 1.66, and the CST group (*M* = 0.19, SD = 0.20), *t*(38) = 3.65, *p* < 0.001, *d* = 1.15. The sTST group also had a significantly higher *c* than the CST group, *t*(38) = 2.73, *p* = 0.009, *d* = 0.86.

**Fig. 3:**
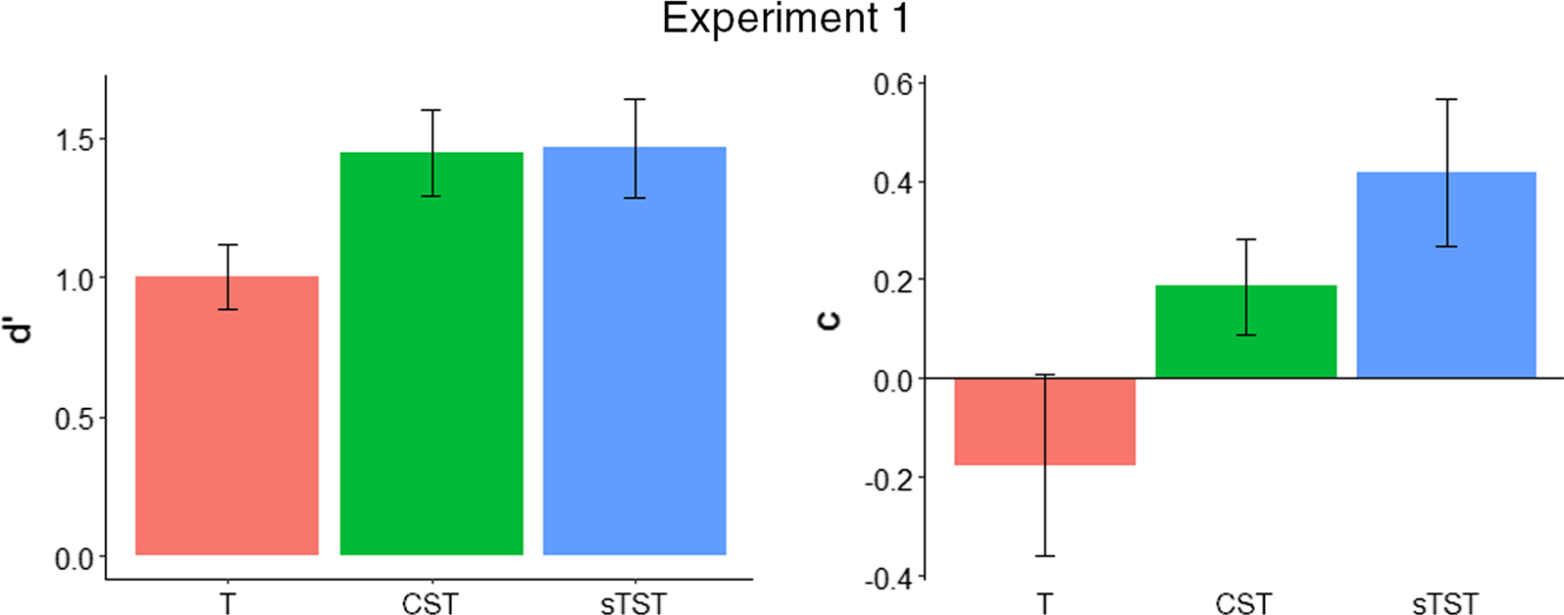
*d′* and *c* for the target-based search training (T), standard targetless search training (sTST) and combined search training (CST) groups in Experiment 1 (error bars show 95% CIs)

**Fig. 4:**
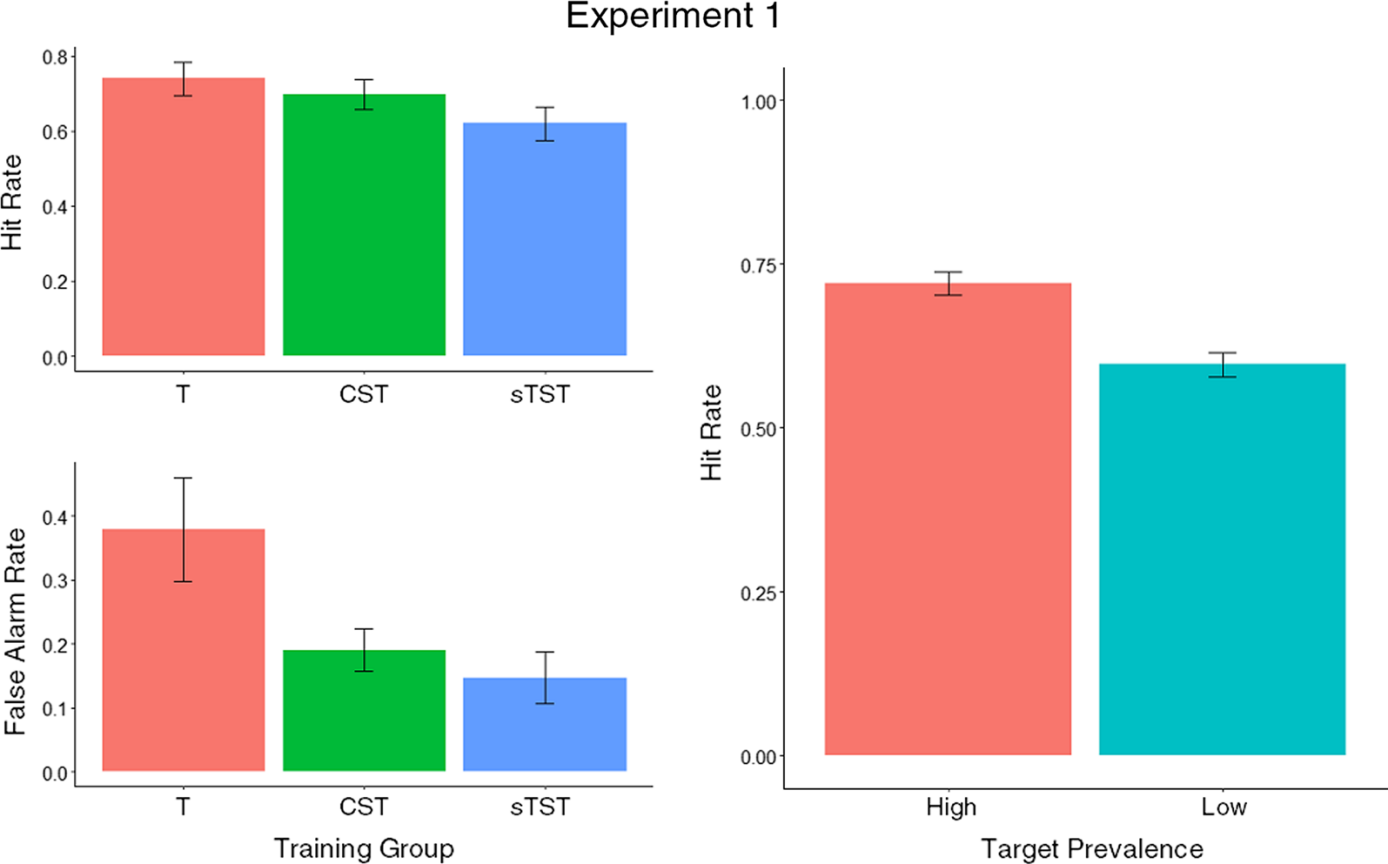
Hit rate and false alarm rate by training group, prevalence and overlap conditions corresponding to LMM analysis for Experiment 1 (target-based search training [T], standard targetless search training [sTST] and combined search training [CST]), error bars show 95% CIs)

Combined results from all experiments are shown in Table 1 and for Experiment 1 in Figs. 3 and 4. In these results, a negative value of *c* indicates a more liberal response criterion (lower threshold to respond target-present) and a positive value of *c* indicates a more conservative response criterion (higher threshold to respond target-present).

**Table 1:** Mean Hit Rates and False Alarm Rates for the Target-based Search Training, Standard Targetless Search Training (sTST) and Combined Search Training (CST) Groups in Experiment 1, Semantic and Alphabetic Training Groups in Experiment 2, Fixed Reward (FR) and Performance-contingent Reward (PR) Groups in Experiment 3 and the Enhanced Targetless Search Training (ETST) and Practice Only (PO) Groups in Experiment 4 (overlap shown where relevant for Experiments 3 and 4; standard deviations shown in brackets)

Experiment	Training group	Hit rate		False alarm rate	
		Overlap	Non-overlap	Overlap	Non-overlap
Experiment 1	Target-based	–	0.74 (0.10)	–	0.38 (0.17)
	sTST	–	0.62 (0.10)	–	0.15 (0.09)
	CST	–	0.70 (0.09)	–	0.19 (0.07)
Experiment 2	Semantic	–	0.61 (0.14)	–	0.14 (0.09)
	Alphabetic	–	0.61 (0.12)	–	0.18 (0.12)
Experiment 3	FR	0.40 (0.05)	0.67 (0.05)	0.22 (0.08)	0.23 (0.08)
	PR	0.42 (0.07)	0.67 (0.07)	0.22 (0.10)	0.22 (0.10)
Experiment 4	ETST	0.44 (0.07)	0.81 (0.07)	0.15 (0.05)	0.14 (0.05)
	PO	0.41 (0.07)	0.74 (0.07)	0.17 (0.08)	0.15 (0.08)

**Fig. 5:**
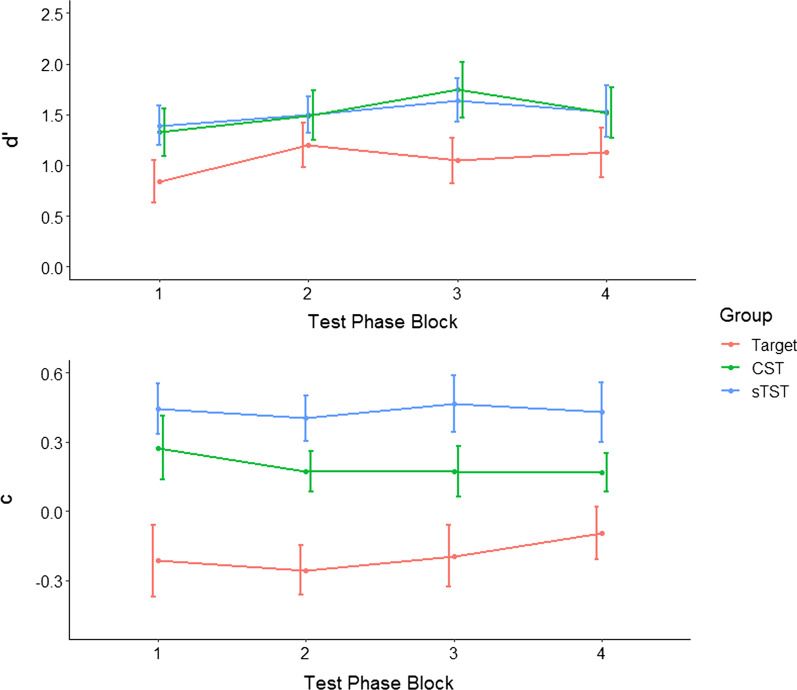
Sensitivity (*d′*) and criterion (*c*) by test phase block (the test phase split into four equal blocks of 42 trials to indicate change over time) and training group for Experiment 1, error bars show 95% CIs)

To examine how the effects of training group changed over the time during the test phase, we split the test phase into four equal blocks of 42 trials and plotted *d′* and *c* for each of these (Fig. 5). Visual inspection of this plot shows that both measures remained generally consistent over time, with *d′* levelling off after the first block for all three groups, suggesting that participants adapted to the task demands relatively rapidly and the effects of training did not shift over time. This interpretation was borne out in statistical analysis (two separate two-way mixed ANOVAs with test phase block [1,2,3,4] and training group [target-based, CST, sTST] as factors and *d′* and *c* as dependent variables), which showed effects of training group on both *d′*, *F*(2,57) = 13.36, *p* < 0.001, *η*_*G*_^2^ = 0.15, and *c*, *F*(2,57) = 17.46, *p* < 0.001, *η*_*G*_^2^ = 0.30, and an effect of test phase block on *d′*, *F*(3,177) = 3.84, *p*_*G-G*corrected_ = 0.012, *η*_*G*_^2^ = 0.04. There were no statistically significant interactions between test phase block and training group, *Fs* < 0.90, suggesting that training effects remained consistent over time and that group differences were not due to strategy carryover effects.

To further characterise task performance between groups, we analysed hit rate and false alarm rate using binomial generalised linear mixed-effects models (GLMMs, see Table 2). All responses were entered into the models as binary values indicating whether or not the response was a hit/miss or a correct rejection/false alarm). Standard ‘treatment’ group comparisons were used such that the sTST and CST groups were each compared with the target-based search training group. All models included participant as a random factor, and in all cases, model fitting started with a full set of interactions and iterated through progressively simpler variants until reaching the best-fitting model (any models that failed to converge were excluded). The results of the models show that there were significant effects of training group on hit rate and false alarm rate, specifically that the target-based training group has a higher hit rate than the sTST group and both the sTST and CST groups had lower false alarm rates than the target-based training group. These results also show that low prevalence target categories were associated with a lower hit rate than high prevalence categories.

**Table 2:** Generalised linear mixed-effects models comparing hit rate and false alarm rate in the standard (sTST) and combined search training (CST) groups with the target-based search training group (standard errors in brackets)

	Hit rate	False alarm rate
sTST	− 0.591***	− 1.370***
	(0.143)	(0.215)
CST	− 0.224	− 0.986***
	(0.144)	(0.211)
Target Prevalence	− 0.578***	−
	(0.068)	
Intercept	1.261***	− 0.529***
	(0.105)	(0.147)
Observations	5,003	5,001
Log Likelihood	− 3,014.867	− 2,515.079
Akaike Inf. Crit	6,039.734	5,038.158
Bayesian Inf. Crit	6,072.323	5,064.228

***p<0.001

### Discussion

Our analysis supported our prediction that participants who received sTST training would be better able to detect targets in the baggage search task (at least in terms of *d′*), but further analysis revealed more nuanced differences among the three training conditions. The higher *d′* scores for those who received sTST and CST training, relative to target-based training, demonstrated that learning about non-targets (at least as much as about targets) did benefit target detection. However, our analysis of criterion differences between the training groups revealed that both the target-based training group and the sTST group were biased towards responding to say that bags contained the type of item they had viewed during training (i.e. the target-based group were biased towards responding target-present and the sTST group target-absent). These differences can also be characterised in terms of the hit rate and false alarm data. While participants who received sTST demonstrated significantly lower hit and false alarm rates than participants who received target-based training, participants who received CST demonstrated a statistically equivalent hit rate to target-based training participants, but also a significantly lower false alarm rate. As predicted, we also observed a prevalence effect in the typical direction, that is to say that lower prevalence target categories were associated with a lower hit rate than higher prevalence target categories.

Together these findings suggest that training that focuses on non-target recognition facilitates the detection of novel targets in a simulated baggage screening task. Contrary to our predictions, rather than pushing participants towards a target-based search strategy, training that focused equally on target and non-target recognition reduced the bias present in the other training conditions for responding consistent with training (we explore the potential benefits of equal threat and safe focus in training further in Experiment 4). Experiment 1 provides evidence that training safe item recognition is an effective approach and identifies important limitations involved in this. Experiment 2 builds on these findings, and previous studies of distractor templates, by aiming to determine whether grouping training stimuli together into superordinate semantic categories can improve target detection.

## Experiment 2

Previous studies of distractor templates have indicated that non-targets are primarily represented in terms of their semantic features rather than their visual features (Daffron & Davis, 2015, 2016), this is in contrast to target templates where visual features are prioritised (Godwin et al., 2014). In Experiment 2 we investigated whether semantic grouping of safe items into superordinate categories during training could benefit target detection relative to an arbitrary alphabetic grouping.

We predicted that semantic grouping of safe item categories would allow participants to more effectively reject safe items by developing distractor templates that included broad semantic features common to multiple specific subordinate categories. We anticipate that a benefit might result from the need to maintain fewer distractor templates if safe items from multiple subordinate categories can be effectively rejected according to a single superordinate template for rejection. Finally, we again predicted that we would observe a standard prevalence effect on hit rate as in Experiment 1.

### Method

#### Participants

Fifty participants (35 females, 15 males; *M*_age_ = 22.90 years, SD = 4.96) were recruited and randomly allocated to one of two training groups of equal size. Participant recruitment and reimbursement were the same as for Experiment 1.

#### Apparatus and stimuli

The apparatus and stimuli used in Experiment 2 were the same as in Experiment 1.

#### Design and procedure

The design and procedure of Experiment 2, including the familiarisation phase and the testing phase, were the same as Experiment 1, but a different training phase was used. In Experiment 2, participants were randomly assigned to one of two training groups, one of which received a training phase that included semantic categorisation and one of which received a training phase that included alphabetic categorisation. For both groups, the training phase consisted of 196 trials in total. The first 98 trials involved categorising safe items, as in Experiment 1, and the second 98 trials were dependent upon the training group and involved either semantic or alphabetic categorisation.

The semantic and alphabetic categorisation trials were similar to the more specific categorisation task used in first 98 training trials, however, instead of 14 specific object categories presented on the left of the screen, only three superordinate category labels were presented. Participants were asked to group objects into these new categories and click the category in which they had grouped the current object. For the semantic training group, the three categories were: ‘wearables’, ‘multimedia’ and ‘household objects’, and for the alphabetic training group, the three categories were based on the first letter of each object’s name: ‘A-I’,’ J-Q’,’ R-Z’. Feedback was provided for all training trials in the same was as in Experiment 1.

### Results

Analysis of sensitivity (*d′*) and criterion (*c*) revealed no significant differences between the semantic and alphabetic training groups, with additional Bayesian analysis favouring the null hypothesis.

A between-subjects *t* test on *d′* scores was carried out between the semantic (*M* = 1.48, SD = 0.30) and alphabetic (*M* = 1.31, SD = 0.38) training groups. There was no significant difference between groups, *t*(48) = 1.69, *p* = 0.098. We also carried out a two-sided Jeffreys–Zellner–Siow Bayesian *t* test, which showed that the data were 1.12 (BF_01_) times more likely under the null than alternative hypothesis, providing weak evidence against the effect of training group on *d′*.

A between-subjects *t* test on *c* scores was carried out between the semantic (*M* = 0.47, SD = 0.45) and alphabetic (*M* = 0.37, SD = 0.36) training groups. There was no significant difference between groups, *t*(48) = 0.87, *p* = 0.391. A two-sided Jeffreys–Zellner–Siow Bayesian *t* test, which showed that the data were 2.60 (BF_01_) times more likely under the null than alternative hypothesis, providing weak evidence against the effect of training group on *c.*

**Fig. 6:**
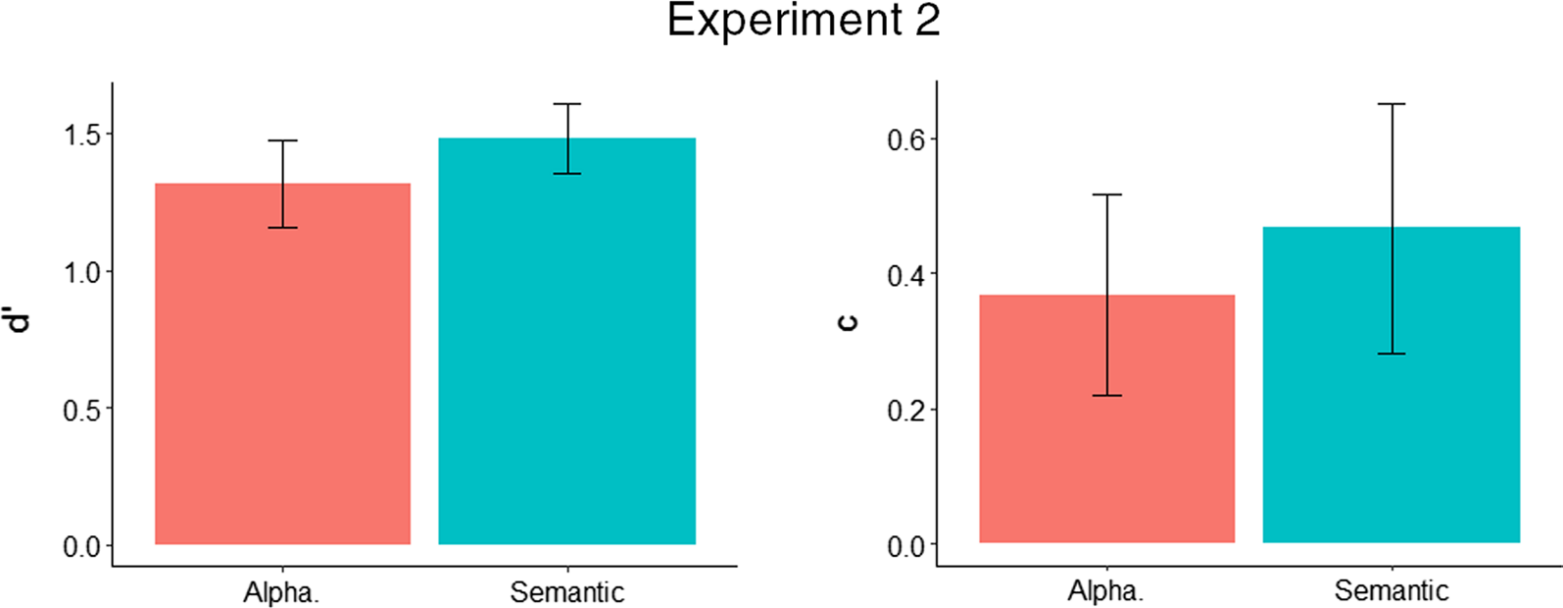
* d′* and *c* for the semantic and alphabetic training groups in Experiment 2 (error bars show 95% CIs)

Given the lack of effects in *d′* and *c* (see Fig. 6), no further follow-up analyses were carried out, with the exception of a GLMM to examine the effect of target prevalence on hit rate (see Table 3).

**Table 3:** Generalised linear mixed-effects model comparing hit rate between high and low target prevalence categories in Experiment 2 (standard errors in brackets)

	Hit rate
Target prevalence	− 0.448***
	(0.072)
Intercept	0.583***
	(0.084)
Observations	4,200
Log likelihood	− 2,716.736
Akaike Inf. Crit	5,439.472
Bayesian Inf. Crit	5,458.501

***p<0.001

### Discussion

Contrary to our predictions, Experiment 2 provided no evidence that training participants to group safe items into semantically-related superordinate categories afforded any benefit to threat detection in the simulated baggage search task relative to an alphabetic grouping control training group. It should also be noted that our Bayesian analysis of *d′* showed only very weak evidence in favour of the null hypothesis, a somewhat ambiguous result which is difficult to interpret, however, it does not rule out the possibility that an alternative semantic grouping could have a larger effect. However, the results of Experiment 2 did serve to replicate the findings of Experiment 1 with respect to the *d′* and *c* observed in the sTST group.

Indeed, *d′* and *c* scores for both training groups in Experiment 2 were similar to those of the sTST group in Experiment 1, which followed a training procedure that was similar but did not include superordinate grouping. These results suggest that participants did not develop broad distractor templates that could facilitate the rejection of multiple semantically-related object categories in response to training, or that they did, but that participants in the alphabetic condition grouped items in a similar way, contrary to their instructions.

## Experiment 3

Experiments 1 and 2 have provided evidence that training novice participants to recognise safe items and develop distractor templates facilitates the detection of threats in a simulated baggage search task. Experiment 3 aimed to test whether the effect of this training can be enhanced with additional motivation and includes a training group that received performance-contingent rewards (contrasted with a fixed reward group). While the stakes involved in a laboratory experiment will never be as high as those involved in airport security screening, we wished to provide participants with at least some additional reason to really try their best in our task.

Experiment 3 also introduced a key element of real-world baggage search that was missing from Experiments 1 and 2, which is a greater degree of spatial overlap within our testing stimuli. In the variant of the baggage search task used here, a proportion of the testing stimuli incorporated a greater degree of spatial overlap in to better simulate the real-world baggage search environment (Donnelly et al., 2019).

We predicted that participants who received performance-contingent rewards would be more motivated and engaged with the test task and that this would be reflected in higher *d′* prime scores and a reduced bias towards responding ‘safe’ (lower *c*) than the fixed reward group. In previous results, participants demonstrated a recognition bias for trained items, with TST participants more likely to respond target-absent, we anticipated that greater task engagement associated with performance-contingent rewards might reduce this bias. We also predicted that all participants would demonstrate impaired target detection in test trials that included more item overlap and that this would be reflected in lower hit rates and higher false alarm rates. We predicted a typical effect of the relative prevalence of different target categories on hit rate, as in Experiments 1 and 2.

### Method

#### Participants

Fifty participants (36 females, 14 males; *M*_age_ = 23.90 years, SD = 4.51) were recruited and randomly allocated to one of two groups of equal size. Participants in one group received reimbursement that was contingent upon their testing phase performance (minimum payment of £6 and one additional pound for reaching 55%, 60%, 65% and 70% accuracy in the testing phase, up to a maximum total payment of £10) and the other group received a fixed amount (£8). Participant recruitment was the same as for Experiment 1.

##### Apparatus and Stimuli.

**Fig. 7:**
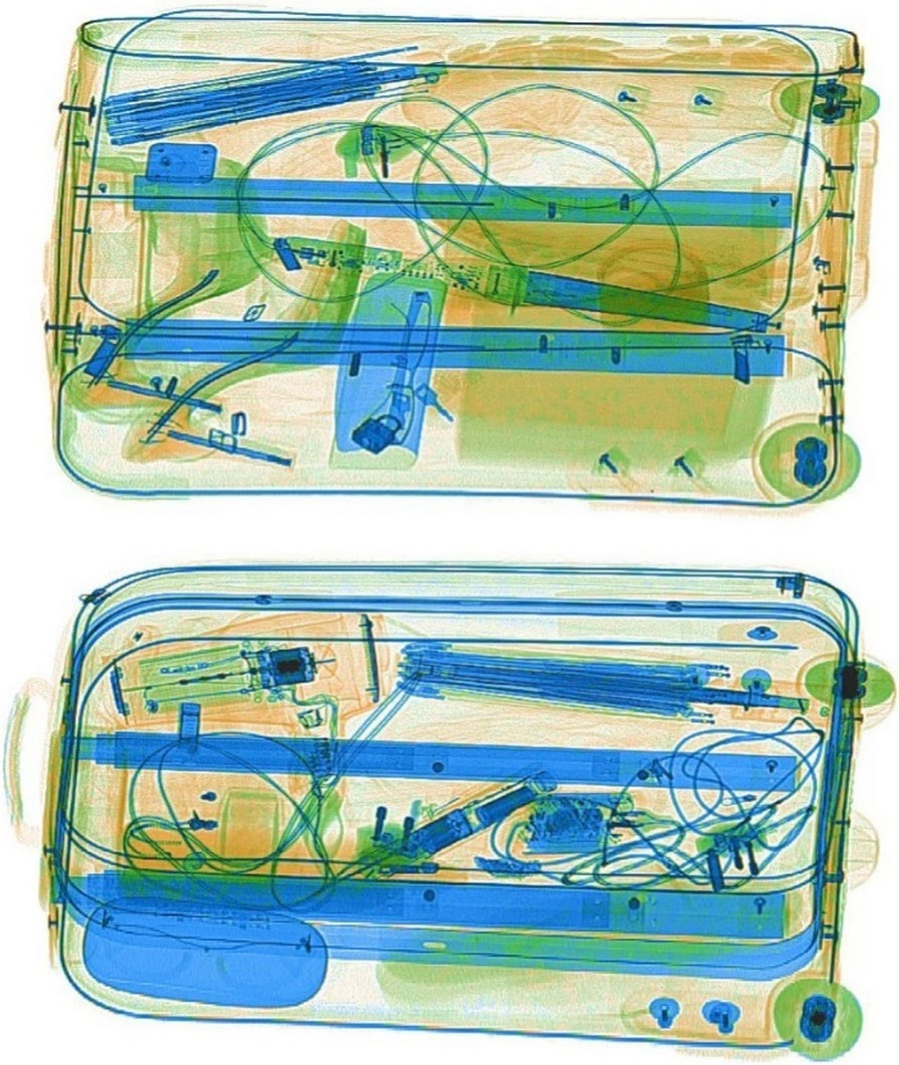
Stimuli from the CaSePIX image library (not used in the present study) illustrative of the levels of spatial overlap present in stimuli with greater overlap used in the test phases of Experiments 3 and 4

The apparatus and stimuli were the same as Experiment 1, with the exception of changes to the testing phase stimuli. We used SimFox to generate 56 new testing phase bag stimuli by selecting 28 existing threat bags and 28 safe bags (from the testing phase stimulus set developed for Experiment 1) and increasing the overlap between objects within the bags. It was not possible to quantify and compare levels of overlap between different bag stimuli containing different sets of objects, but two of the authors (AM and MGP) independently reviewed all stimuli and agreed on their suitability. While we are unable to share the stimuli used at test in this experiment for licensing reasons, Fig. 7 shows stimuli from our own CaSePIX image library which are illustrative of the higher levels of spatial overlap present in the test phases of Experiments 3 and 4.

#### Design and procedure

The design and procedure were the same as Experiment 2 with some exceptions as follows. Participants were randomly allocated to one of two groups: performance-contingent reward group and the fixed reward group. While fixed reward participants received £8 irrespective of their performance at test, the performance-contingent reward group received £6 base rate and up to £4 more—one additional pound for surpassing each of the four accuracy thresholds at test (listed above). In the test phase, the trials involving bags with additional overlap were randomly interleaved with standard trials.

Participants were informed of their group allocation. Fixed reward participants were told that they would receive £8 and performance-contingent reward participants were told that they would receive at least £6 with up to £4 more depending on their performance (they were not informed of the specific performance thresholds). Participants were informed that the overall target prevalence level was 50% to minimise the possibility that the effect of reward was masked by an inappropriate overall response criterion. Participants in the performance-contingent reward group received feedback on their performance (percentage correct) and their reward at the end of the test phase.

### Results

Analysis of sensitivity (*d′*) and criterion (*c*) revealed no significant differences between the fixed and performance-contingent reward groups, with additional Bayesian analysis favouring the null hypothesis.

A between-subjects *t* test on *d′* scores was carried out between the fixed reward (*M* = 0.98, SD = 0.21) and performance-contingent reward (*M* = 1.03, SD = 0.28) groups. There was no significant difference between groups, *t*(48) = 0.66, *p* = 0.510. We again carried out a two-sided Jeffreys–Zellner–Siow Bayesian *t* test, which showed that the data were 2.95 (BF_01_) times more likely under the null than alternative hypothesis, providing weak evidence against the effect of training group on *d′*.

**Fig. 8:**
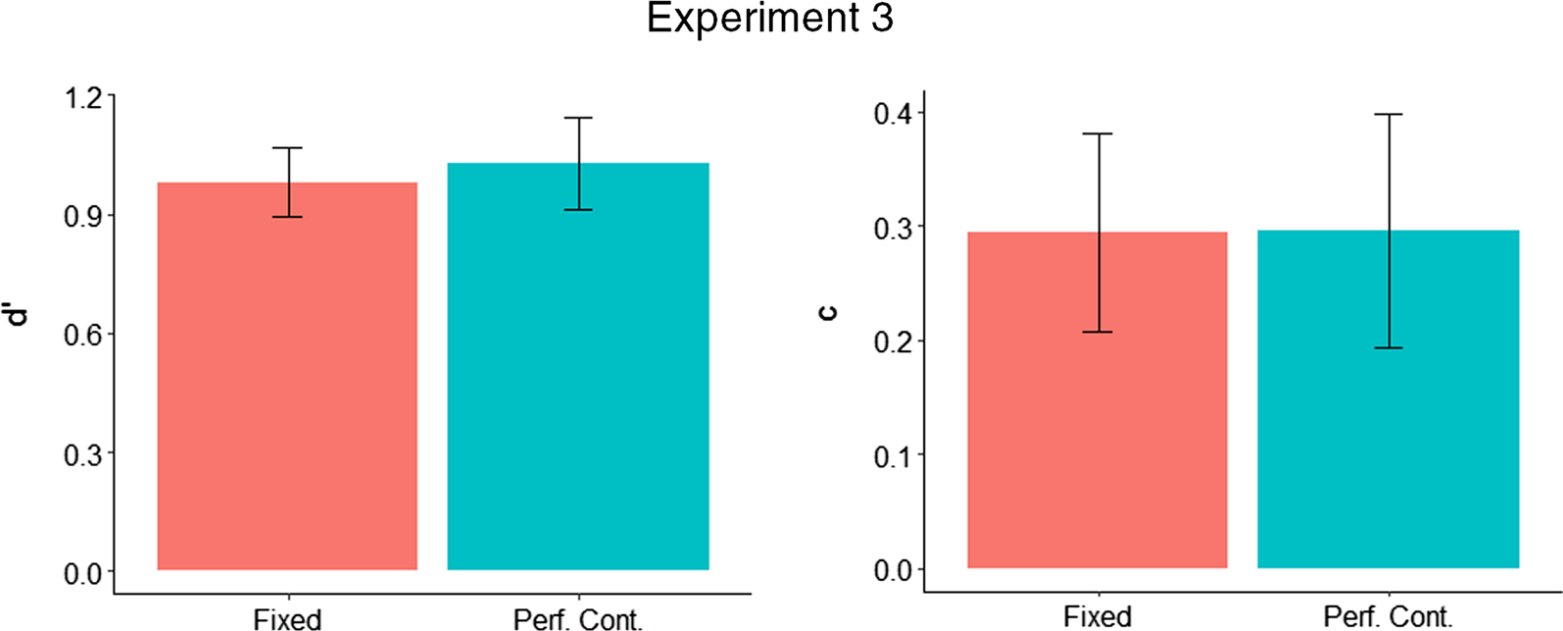
* d′* and *c* for the fixed and performance-contingent reward groups in Experiment 3 (error bars show 95% CIs)

A between-subjects *t* test on *c* scores was carried out between the fixed reward (*M* = 0.29, SD = 0.21) and performance-contingent reward (*M* = 0.30, SD = 0.25) groups. There was no significant difference between groups, *t*(48) = 0.03, *p* = 0.979. A two-sided Jeffreys–Zellner–Siow Bayesian *t* test, which showed that the data were 3.54 (BF_01_) times more likely under the null than alternative hypothesis, providing positive evidence against the effect of training group on *c*. See Fig. 8 for *d′* and *c* results from Experiment 3.

**Fig. 9:**
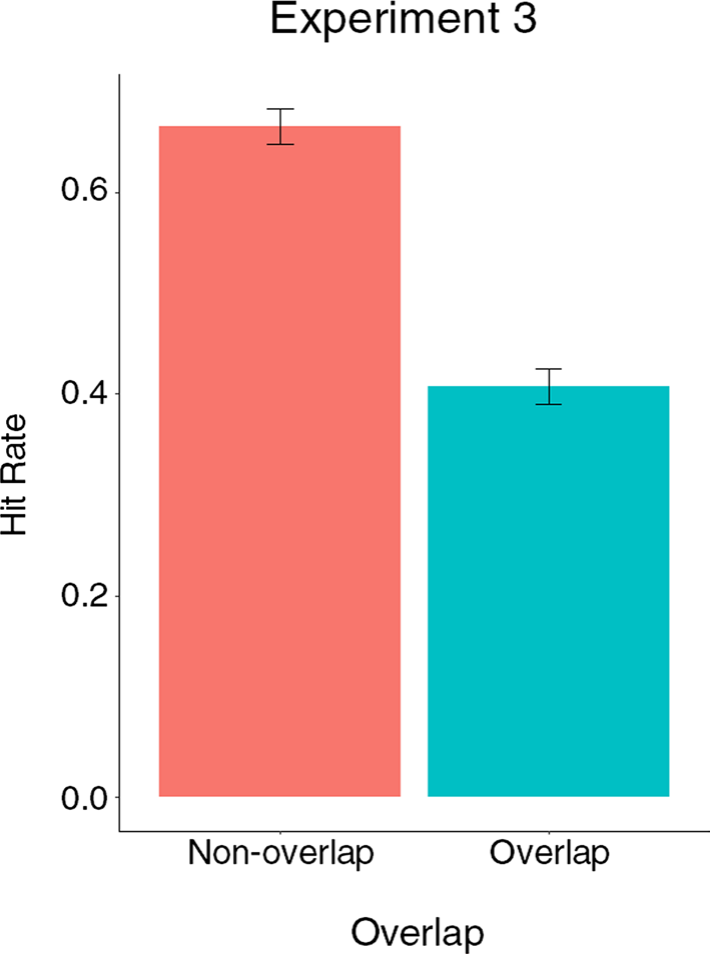
Hit rate and false alarm rate by overlap corresponding to LMM analysis for Experiment 3 (error bars show 95% CIs)

To test the influence of overlap in displays at the training group level, we followed the same process as for Experiment 1 and analysed hit rate and false alarm rate using binomial generalised linear mixed-effects models (GLMMs, see Table 4, results summarised in Fig. 9). In this case overlap was also included as a factor. A prevalence effect on hit rate was not present, likely because of the inclusion of overlap trials. There were no effects of training group, but hit rate was significantly lower overall on overlap trials (overlap did not interact with training group). The best fitting model of false alarm rate was an intercept only model, as neither group nor overlap were significant predictors of false alarm rate.

**Table 4:** Generalised linear mixed-effects models comparing hit rate and false alarm rate including training group and overlap as factors in Experiment 3 (standard errors in brackets)

	Hit rate	False alarm rate
Training Group	–	–
Target Prevalence	–	–
Overlap	− 1.096***	–
	(0.068)	
Intercept	0.710***	− 1.294***
	(0.062)	(0.066)
Observations	4,284	4,284
Log likelihood	− 2,758.000	− 2,252.913
Akaike Inf. Crit	5,522.000	4,509.827
Bayesian Inf. Crit	5,541.088	4,522.552

***p<0.001

### Discussion

While Experiment 3 provided no evidence of a difference in performance between the two reward groups, it did show that test trials which included more overlap were associated with a significantly lower hit rate (compared to other test trials). While it is possible that providing performance-contingent reward was an ineffective motivator, it is more likely, given the reduced *d′* and hit rate relative to Experiments 1 and 2, that the inclusion of overlap trials in the test phase was sufficiently challenging that improved motivation could not provide additional performance benefits within the time allowed by the experiment. We acknowledge that our Bayesian analyses of *d′* and *c* showed only weak evidence in favour of the null hypotheses, however, these results do not rule out the possibility that performance-contingent rewards could improve performance, either with a greater reward or less challenging stimuli.

Oddly there was no effect of target prevalence as predicted and as observed in Experiments 1 and 2. At first glance, this did not appear to be due to overlap or the training groups in the present experiment, as no interaction between target prevalence and either of these factors was observed. However, further investigation suggested that the lack of a clear interaction, or indeed a main effect of target prevalence, may be due to the increased variability in hit rate associated with higher levels of overlap.

The additional challenges that overlap poses, in terms of impeding item segmentation, boundary detection and the use of colour, are significant and these results suggest that our current training procedure has limited effectiveness in the face of these challenges. In Experiment 4 we introduce a new training procedure with the aim of attaining a similar level of target detection performance to that observed in the CST group in Experiment 1, but with stimuli that involve higher levels of overlap.

## Experiment 4

Experiment 4 aimed to test the effectiveness of an enhanced TST procedure (ETST), developed and optimised based on the findings from all three previous experiments. ETST incorporated threat and safe item training (as in the CST condition of Experiment 1), item categorisation involving three items presented together (to provide a basic introduction to the challenges of item segmentation), and practice test trials with feedback (giving participants the opportunity to adjust their expectations). As we wished to test the overall effectiveness of ETST rather than to test it against another training procedure, we provided another group of participants with an equivalent duration of practice (test trials but with feedback) to provide a simple baseline for comparison.

We expected that a longer training duration, with training occurring on two days, would allow more time for the development and consolidation of effective distractor templates. Compared to the standard categorisation trials, the inclusion of three-item categorisation and practice trials were intended to facilitate the identification of multiple non-targets together, rather than in isolation. We predicted that participants who received ETST would be better able to detect threats during the test phase than participants who only received practice and that this would be evident in terms of *d′*, hit rate and false alarm rate.

### Method

#### Participants

Thirty-two participants (19 females, 13 males; *M*_age_ = 24.13 years, SD = 5.19) were recruited and randomly allocated to one of two training groups of equal size. Participants were reimbursed £25 for completing the experiment. Participant recruitment was the same as for Experiment 1.

#### Apparatus and stimuli

The apparatus and stimuli were the same as for Experiment 3, with the exception of stimuli in the new three-item categorisation training block. We used SimFox to generate 130 new three-item bag stimuli. Each of was a simplified bag stimulus containing only three safe items from our existing categories. No one of these stimuli contained more than a single example of a given category of safe item (see Additional file 1: Supplementary Tables S1–S5 for further details).

#### Design and procedure

**Fig. 10:**
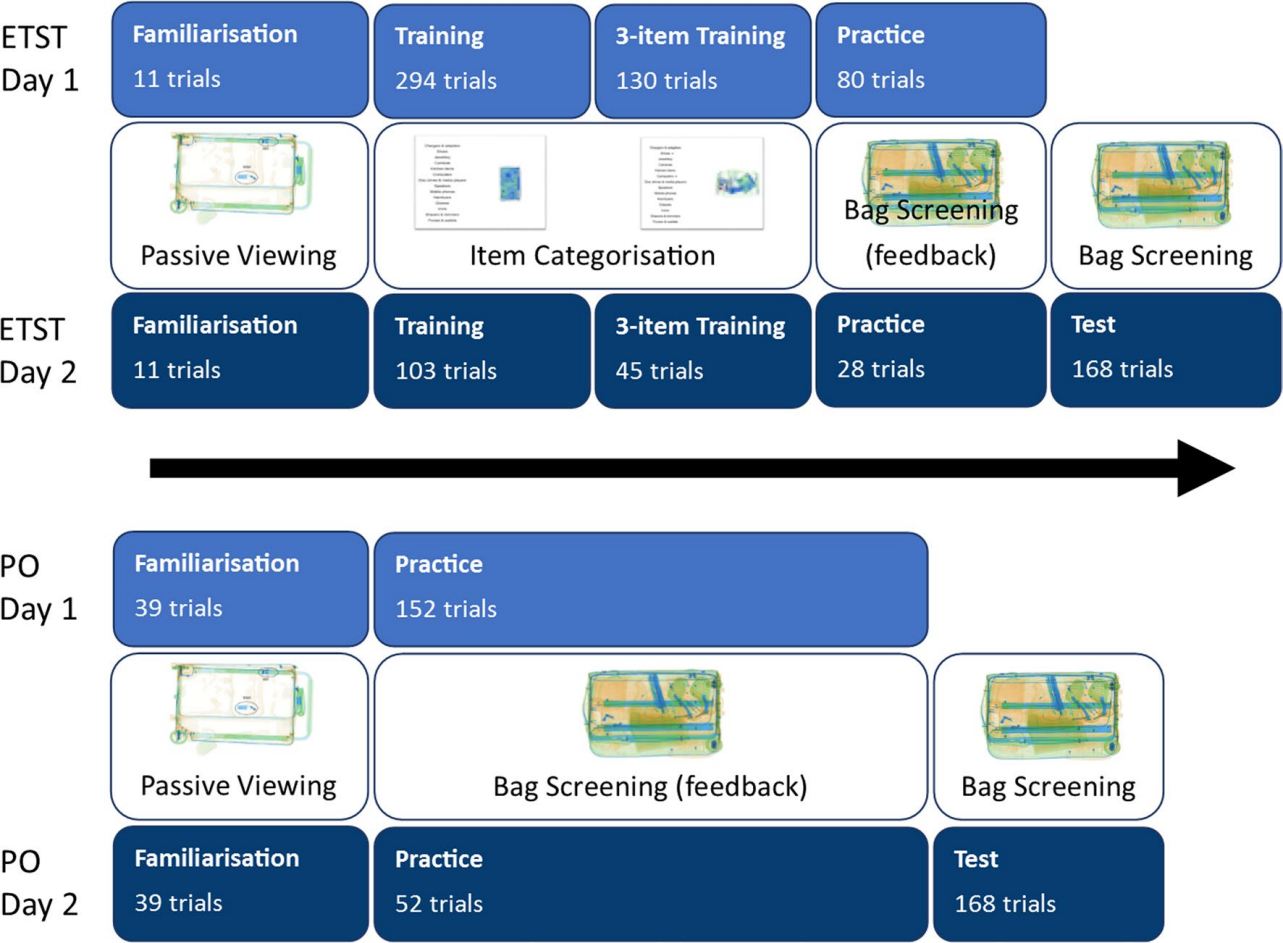
Outline procedures for Experiment 4

Participants were randomly allocated to one of two groups: the ETST group or the practice only (PO) group. Both groups completed two training sessions on separate days, with a test at the end of the session on the second day. The second session was never more than two days after the first. The procedure for each group is shown in Fig. 10 (figure includes stimuli not used in the experiment shown for illustrative purposes only).

The PO group completed an amended familiarisation phase at the start of both sessions, which included a pair of examples from each of the threat and safe item categories (28 additional trials following the 11 familiarisation trials used in the previous experiments). This was to provide participants in the PO group with some basic level of familiarity with the appearance of the items that would appear in bags during the practice and test phases. The PO group completed 152 practice trials in session one and 52 in session two. Practice trials in this experiment were identical to test phase trials, with the exception that feedback was given following the response.

The ETST group completed a standard familiarisation phase at the start of both sessions. In session one, this was followed by 294 single safe item categorisation trials (as in previous experiments), 130 three safe item categorisation trials and 80 practice trials. In session two, the ETST group completed the same phases in the same order, but with 103 single item trials, 45 three item trials and 28 practice trials. The three item categorisation trials were similar to the single item categorisation trials used in this and previous experiments, with the exception that instead of needing to click a single category, participants were required to click three categories to make a complete response. In each three item trial, when a category was clicked it was marked with an arrow and after three categories were clicked, feedback was shown and the trial ended as for the single item trials (no time limit).

Both groups completed a final test phase at the end of the second session was identical to that in Experiment 3 with the exception that participants were not informed about target prevalence. While the ETST completed more trials (across all training blocks) than the PO group, both groups were matched for total time on task across all days.

### Results

Analysis of sensitivity (*d′*) and criterion (*c*) revealed marginally higher *d′* scores for the ETST group over the PO group and no significant difference in *c*, with this result driven by a significantly higher hit rate in the ETST group relative to the PO group.

A between-subjects *t* test on *d′* scores was carried out between the ETST (*M* = 1.62, SD = 0.39) and PO (*M* = 1.37, SD = 0.34) training groups. There was a marginal difference between groups, *t*(30) = 1.87, *p* = 0.072.

**Fig. 11:**
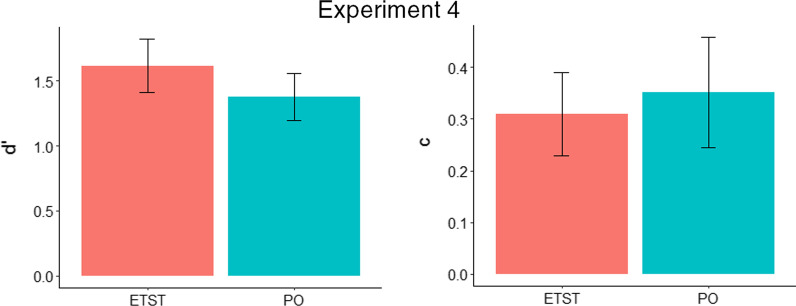
* d′* and *c* for the enhanced targetless search training (ETST) and practice only (PO) groups in Experiment 4 (error bars show 95% CIs)

A between-subjects *t* test on *c* scores was carried out between the ETST (*M* = 0.31, SD = 0.15) and PO (*M* = 0.35, SD = 0.20) training groups. The difference between groups was non-significant, *t*(30) = 0.67, *p* = 0.506. See Fig. 11 for *d′* and *c* results.

**Fig. 12:**
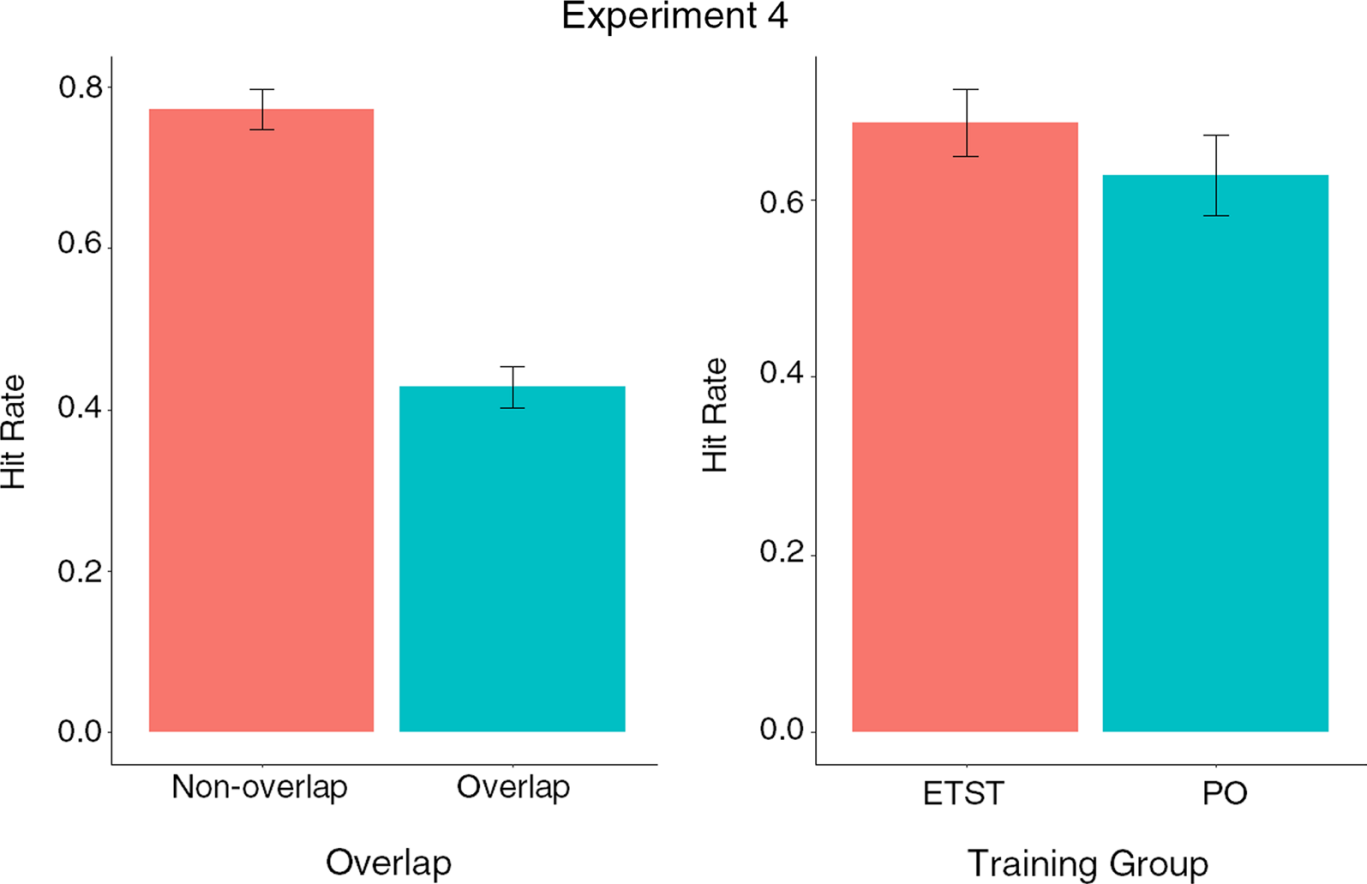
Hit rate and false alarm rate by training group and overlap conditions corresponding to LMM analysis for Experiment 4 (enhanced targetless search training [ETST] and practice only [PO], error bars show 95% CIs)

To further characterise task performance between groups, we followed the same process as for Experiment 1 and analysed hit rate and false alarm rate using binomial generalised linear mixed-effects models (GLMMs, see Table 5). Overlap was also included as a factor, as well as prevalence and training group as per the analysis of the previous experiments. The ETST group had a significantly higher hit rate than the PO group (see Table 1 and Fig. 12). Accuracy was significantly lower overall on overlap trials and overlap did not interact with training group. We once again observed a conventional prevalence effect on hit rate, as in Experiments 1 and 2, and the effects of prevalence and overlap interacted. The best fitting model of false alarm rate was an intercept only model, with neither group nor overlap as significant predictors.

**Table 5:** Generalised linear mixed-effects models comparing hit rate and false alarm rate including training group and overlap as factors in Experiment 4 (standard errors in brackets)

	Hit rate	False alarm rate
Training group	− 0.373*	–
	(0.171)	
Overlap	− 1.827***	–
	(0.164)	
Target Prevalence	− 0.566**	–
	(0.205)	
Training Group × Overlap	0.408 (0.228)	–
Training Group × Target Prevalence	− 0.223 (0.276)	–
Overlap × Target Prevalence	0.588* (0.281)	–
Training Group × Overlap × Target Prevalence	− 0.150 (0.390)	–
Intercept	1.587***	− 1.793***
	(0.125)	(0.091)
Observations	2,688	2,688
Log Likelihood	− 1,540.954	− 1,123.370
Akaike Inf. Crit	3,099.907	2,250.741
Bayesian Inf. Crit	3,152.976	2,262.534

*p<0.05; **p<0.01; ***p<0.001

### Discussion

The ETST group demonstrated a marginally higher *d′* score than the PO group. Examining the hit and false alarm rates revealed that this was driven by the ETST group having a significantly higher overall hit rate than the PO group with no difference in overall false alarm rate. Compared with performance in the CST condition of Experiment 1 and, excepting overlap, the new ETST procedure in this experiment was associated with a low false alarm rate and high hit rate.

Neither the additional training time nor opportunity for consolidation afforded by multiple training sessions on separate days allowed participants in either the ETST or PO groups to bring their hit rate in overlap trials closer to that of non-overlap trials and the difference in hit rates was similar to that observed in Experiment 3. Further, as in Experiment 3, overlap did not interact with training group, neither practice nor ETST provided a clear benefit to dealing with overlapping items. However, unlike in Experiment 3, target prevalence and overlap interacted, such that the effect of prevalence on hit rate, similar to that observed in Experiments 1 and 2, was primarily driven by performance in trials with lower levels of overlap. This is consistent with higher levels of overlap interfering with object identification, effectively lowering target prevalence across categories in high overlap trials because objects in these trials are less likely to be recognised and identified. The presence of a significant interaction between prevalence and overlap in the present experiment, but not in Experiment 3, may be due to a reduction in hit rate variability associated with the enhanced training provided in the present experiment. Presumably participants would have been better able to learn about the ways in which overlap can change objects’ appearance with more experience of items presented together in overlapping arrangements.

Despite these difficulties, the higher hit rate for the ETST group relative to the PO group suggests that participants do benefit from training designed to introduce objects and simple examples of how they might overlap spatially, rather than just being presented with challenging full bags from the start. In the context of a training procedure that is still short in length relative to current professional procedures and a challenging test phase with spatially overlapping objects, the improvement in hit rate relative to practice is remarkable.

## General discussion

The primary aim of the present study was to investigate whether basic principles of distractor templates and suppression could be used to successfully train novice X-ray baggage screeners. To achieve this, we developed and tested a targetless search training procedure which focussed on training participants to utilise broad categorical distractor templates to recognise distractors during search. We examined the effects of targetless training on novice screeners in a simulated baggage search task across four experiments, which together sought to determine the effectiveness of this approach relative to target-based training and in terms of well-established challenges involved in baggage search such as those posed by low target prevalence, overlap and heterogenous (and difficult to predict) target categories.

Our targetless search training procedure was designed to train novice screeners to develop broad categorical distractor templates. We trained novice screeners to recognise 14 distinct categories of non-target item, all of which are examples of safe items that are permitted in cabin baggage in the real-world, and then tested their performance in a simulated baggage search task. Together, Experiments 1 through 4 demonstrated that it is possible to search effectively using a set of categorical distractor templates in a simulated baggage screening task. This result can be broken down into three important conclusions about the role of distractor templates in the context of baggage screening and personnel training: (1) using distractor templates improves distractor recognition and target detection sensitivity relative to conventional search focussed on target templates; (2) using distractor templates exclusively results in a recognition bias, but this can be mitigated when supplemented with target training; (3) overlap and low relative target prevalence pose difficulties even for search enhanced by using distractor templates.

Experiment 1 demonstrated that searching using distractor templates resulted in improved target detection sensitivity relative to searching only with target templates. In particular, participants who were trained to use distractor templates demonstrated significantly lower false alarm rates than participants trained to use target templates only. We observed comparable recognition biases in the two groups trained to use only target templates (target-based training) or only distractor templates (sTST), such that both of these groups tended to respond in a way that was consistent with their training, i.e. the target-based training group tended to respond ‘target-present’ and the sTST group tended to respond ‘target-absent’. This bias was not observed to the same extent in the group that was trained to use both target and distractor templates (CST). We expect that the differences in response criterion between the three training groups reflect participants forming their own expectations about threat prevalence in the test phase and that these differences could have been reduced through instructions. Participants in the sTST group exhibited a criterion more in line with the ultra-low prevalence of threats in real-world screening than a 50% prevalence laboratory experiment, which we believe is a practical strength of a TST approach.

In assessing the outcomes of baggage screening, it is tempting to assign priority to hit rates, after all, the consequences of missing an improvised explosive or a weapon would be disastrous. However, hit rates alone can be easily increased by instructions that cause a criterion shift, increasing false alarms at the same time. The reason this is not done in practice is that while high false alarm rates sound benign, due to the many millions of safe bags passing through airports every day (and the comparatively low number of threat bags—almost zero for some threat categories) false alarms can drastically slow the screening process. Controlling false alarm rates while maintaining threat sensitivity is the key challenge facing both human and machine solutions to screening.

It is also important to note, that for all groups in Experiment 1 where threat items were present in training, these items were not used in any of the subsequent test stimuli, but safe items were. This resulted in a set of test stimuli that better represented the challenges of real-world baggage search, including novel threats, homogenous safe item categories and some repetition of common safe items across bags. In the trade-off between experimental control and ecological validity, given the motivation of the present work, we opted to favour the latter. When we analysed performance differences in response to stimuli repeated between training and test we found no differences across training groups, suggesting that our stimulus selection did not confer unequal benefits to specific training groups.

Results from Experiments 2 and 3 provided no evidence that performance-related payments or grouping categories of objects into broader semantic categories can improve performance using targetless search training. Given that semantic grouping *did* enhance performance in previous work using simple photographs of objects against natural scenes (Daffron & Davis, 2015, 2016), further investigation of this possibility may yet be worthwhile.

One clear indication from Experiment 4 was that longer training serves to enhance threat detection, with sensitivity to threats highest in this experiment. While this was not a manipulation of specific interest here, it offers some practical reassurance that screeners can be taught, in a small number of brief training sessions, to detect threats to which they might ordinarily be blind.

The results from Experiment 4 showed a marginal benefit to sensitivity (*d′*) for the ETST group over the practice only group and further analysis revealed that this effect was driven by a higher hit rate for the ETST group. Further, there was a main effect of overlap on hit rate, such that overlap was associated with a lower hit rate. An explanation of these effects is that interleaved trials with additional overlap (amongst standard trials) keeps the response criterion down for all participants, irrespective of training group, due to the challenges involved in object segmentation and identification for this type of trial. While the criterion remains constant however, training group influences sensitivity to some extent despite the difficulties involved in resolving overlapping images, resulting in a higher hit rate for the ETST group. This result suggests that threats are more difficult to resolve, segment and identify than the safe items when these items overlap with other items. If the overlap makes it harder to identify threat items (or indeed any item) and bags contain more safe items than threats, then participants may be more likely to identify a bag as safe because they have been able to recognise at least one safe item.

We observed a large benefit to sensitivity for sTST and CST groups (relative to target-based training) in Experiment 1. In subsequent experiments we observed smaller effects as we attempted to better characterise the benefits of TST. In Experiments 3 and 4, we introduced bags with a high degree of overlap, which were the most difficult for participants to identify (as threat/safe) irrespective of training group. These stimuli are likely representative of densely packed baggage encountered in the real-world which pose a challenge even to professional screeners. However, unlike professional screeners, all participants in the experiments reported here were novices, receiving no more than two short training sessions. We anticipate that the large benefits of targetless training observed with low-overlap stimuli would also be observed in response to high-overlap stimuli if participants were given extended training (multiple sessions over several days), including more opportunities to learn about segmentation, edge detection and how overlap can change the appearance of objects. This is beyond the scope of the present study but should certainly be addressed in future work.

The experiments described here constitute initial evidence that a targetless search technique, based on distractor templates, can improve detection of threats in X-rayed-baggage images by novice screeners, including rare and challenging targets under the types of conditions observed in routine X-ray baggage screening. While this was consistent with pilot work that found similar advantages for targetless search using small arrays of challenging photographic stimuli, we predicted that this should be the case with X-ray stimuli a priori, given difficulty in specifying heterogenous target categories and the much lower prevalence of targets than nontargets in our experiments (a pattern paralleled in the real-world). While the relative benefits of sTST versus CST require further investigation, in practice, it seems unlikely that screeners would have no experience of the appearance of threat items and we expect that something resembling CST would be most likely to be adopted. We speculate that, at its heart, the benefit of TST for baggage screening is that every bag screened affords a rich opportunity to recognise and learn about a range of safe items, in a way that is not possible with threat items (given their ultra-low prevalence). Screeners’ exposure to safe items is vast relative to threat items (even accounting for threats artificially inserted in images) and TST leverages this experience to improve item recognition.

Threats in X-rayed baggage images offer some of the most profoundly challenging but important search tasks faced by human or machine. They are ultra-rare, of hugely diverse appearances and are placed by an adversary to minimise the likelihood of detection. Indeed, the most dangerous of threats is entirely novel, meaning that their appearance is typically very poorly specified and adequate search performance requires long training and significant experience. ‘Targetless’ search training avoids this difficulty by not focussing on searching for threats at all, but rather training screeners to learn and use distractor templates to exclude non-targets from their search. We conclude that the best means of searching for novel or highly diverse threats may be not to search for them at all. To paraphrase a famous, fictional detective—once you have safely excluded all the other items, what you have left, however unusual, must be worthy of scrutiny.

## Supplementary Information

**Additional file 1**. Supplementary Tables S1–S5.

## Data Availability

The datasets generated and/or analysed during the current study were produced using without any involvement from staff from Her Majesty’s Government and are available from the corresponding author on reasonable request.

## References

[CR1] Adamo, S. H., Cain, M. S., & Mitroff, S. R. (2018). Satisfaction at last: Evidence for the ‘satisfaction’ account for multiple-target search errors. *Proceedings Volume 10577, Medical Imaging 2018: Image Perception, Observer Performance, and Technology Assessment 105770A*. 10.1117/12.2293692

[CR2] Ariga A, Kawahara J (2004). The perceptual and cognitive distractor-previewing effect. Journal of Vision.

[CR3] Arita JT, Carlisle NB, Woodman GF (2012). Templates for rejection: Configuring attention to ignore task-irrelevant features. Journal of Experimental Psychology: Human Perception and Performance.

[CR4] Barrett DJK, Zobay O (2014). Attentional control via parallel target-templates in dual-target search. PLoS ONE.

[CR5] Buser D, Sterchi Y, Schwaninger A (2020). Why stop after 20 minutes? Breaks and target prevalence in a 60-minute X-ray baggage screening task. International Journal of Industrial Ergonomics.

[CR6] Cain MS, Adamo SH, Mitroff SR (2013). A taxonomy of errors in multiple-target visual search. Visual Cognition.

[CR7] Chang S, Egeth HE (2019). Enhancement and suppression flexibly guide attention. Psychological Science.

[CR8] Daffron JL, Davis G (2015). Templates for rejection can specify semantic properties of nontargets in natural scenes. Journal of Vision.

[CR9] Daffron JL, Davis G (2016). Target templates specify visual, not semantic, features to guide search: A marked asymmetry between seeking and ignoring. Attention, Perception, and Psychophysics.

[CR10] Donnelly N, Muhl-Richardson A, Godwin H, Cave K (2019). Using eye movements to understand how security screeners search for threats in X-ray baggage. Vision.

[CR11] Fleck MS, Mitroff SR (2007). Rare targets are rarely missed in correctable search. Psychological Science.

[CR12] Fleck, M. S., Samei, E., & Mitroff, S. R. (2010). Generalized ‘Satisfaction of Search’: Adverse influences on dual-target search accuracy. *16*(1), 60–71. 10.1037/a0018629.Generalized 10.1037/a0018629 PMC3653986 20350044

[CR13] Gaspelin N, Leonard CJ, Luck SJ (2015). Direct evidence for active suppression of salient-but-irrelevant sensory inputs. Psychological Science.

[CR14] Geng JJ (2014). Attentional mechanisms of distractor suppression. Current Directions in Psychological Science.

[CR15] Godwin HJ, Hout MC, Menneer T (2014). Visual similarity is stronger than semantic similarity in guiding visual search for numbers. Psychonomic Bulletin & Review.

[CR16] Godwin HJ, Menneer T, Cave KR, Donnelly N (2010). Dual-target search for high and low prevalence X-ray threat targets. Visual Cognition.

[CR17] Godwin HJ, Menneer T, Liversedge SP, Cave KR, Holliman NS, Donnelly N (2017). Adding depth to overlapping displays can improve visual search performance. Journal of Experimental Psychology: Human Perception and Performance.

[CR18] Godwin HJ, Menneer T, Riggs CA, Cave KR, Donnelly N (2015). Perceptual failures in the selection and identification of low-prevalence targets in relative prevalence visual search. Attention, Perception & Psychophysics.

[CR19] Goolsby BA, Suzuki S (2001). Understanding priming of color-singleton search: Roles of attention at encoding and “retrieval”. Perception & Psychophysics.

[CR20] Goolsby BA, Suzuki S (2002). The distractor-color adaptation effect in color-singleton search: What color representation is being adapted?. Journal of Vision.

[CR21] Hättenschwiler N, Mendes M, Schwaninger A (2019). Detecting bombs in X-ray images of hold baggage: 2D versus 3D imaging. Human Factors.

[CR22] Hout MC, Goldinger SD (2015). Target templates: The precision of mental representations affects attentional guidance and decision-making in visual search. Attention, Perception, & Psychophysics.

[CR23] Hout MC, Robbins A, Godwin HJ, Fitzsimmons G, Scarince C (2017). Categorical templates are more useful when features are consistent: Evidence from eye movements during search for societally important vehicles. Attention, Perception, & Psychophysics.

[CR24] McCarley JS, Kramer AF, Wickens CD, Vidoni ED, Boot WR (2004). Visual Skills in Airport-Security Screening. Psychological Science.

[CR25] Menneer T, Barrett DJK, Phillips L, Donnelly N, Cave KR (2007). Costs in searching for two targets: Dividing search across target types could improve airport security screening. Applied Cognitive Psychology.

[CR26] Menneer T, Cave KR, Donnelly N (2009). The cost of search for multiple targets: Effects of practice and target similarity. Journal of Experimental Psychology. Applied.

[CR27] Menneer T, Donnelly N, Godwin HJ, Cave KR (2010). High or low target prevalence increases the dual-target cost in visual search. Journal of Experimental Psychology. Applied.

[CR28] Mitroff SR, Biggs AT (2014). The Ultra-Rare-Item Effect: Visual Search for Exceedingly Rare Items Is Highly Susceptible to Error. Psychological Science.

[CR29] Moher J, Egeth HE (2012). The ignoring paradox: Cueing distractor features leads first to selection, then to inhibition of to-be-ignored items. Attention, Perception, and Psychophysics.

[CR30] Peirce JW (2007). PsychoPy—Psychophysics software in Python. Journal of Neuroscience Methods.

[CR31] Peirce JW (2009). Generating stimuli for neuroscience using PsychoPy. Frontiers in Neuroinformatics.

[CR32] Schwaninger A (2016). Determinants of airport security X-ray screeners’ detection performance. Aviation Security International.

[CR33] Stroud MJ, Menneer T, Cave KR, Donnelly N (2012). Using the dual-target cost to explore the nature of search target representations. Journal of Experimental Psychology: Human Perception and Performance.

[CR34] Vickery TJ, King L, Jiang Y (2005). Setting up the target template in visual search. Journal of Vision.

[CR35] Watson DG, Humphreys GW (1997). Visual marking: Prioritizing selection for new objects by top-down attentional inhibition of old objects. Psychological Review.

[CR36] Watson DG, Humphreys GW (2000). Visual marking: Evidence for inhibition using a probe-dot detection paradigm. Perception & Psychophysics.

[CR37] Wolfe J, Alvarez G, Rosenholtz R, Oliva A, Torralba A, Kuzmova Y, Uhlenhuth M (2008). Search for arbitrary objects in natural scenes is remarkably efficient. Journal of Vision.

[CR38] Wolfe JM, Brunelli DN, Rubinstein J, Horowitz TS (2013). Prevalence effects in newly trained airport checkpoint screeners: Trained observers miss rare targets, too. Journal of Vision.

[CR39] Wolfe JM, Horowitz TS, Wert MJV, Kenner NM, Place SS, Kibbi N (2007). Low target prevalence is a stubborn source of errors in visual search tasks. Journal of Experimental Psychology General.

