# Correction to: Improved X-ray baggage screening sensitivity with ‘targetless’ search training

**DOI:** 10.1186/s41235-021-00327-9

**Published:** 2021-09-03

**Authors:** Alex Muhl‑Richardson, Maximilian G. Parker, Sergio A. Recio, Maria Tortosa‑Molina, Jennifer L. Daffron, Greg J. Davis

**Affiliations:** Department of Psychology, University of Cambridge, Downing Street, Cambridge, CB2 3EB UK

## Correction to: Cogn. Research (2021) 6:33 https://doi.org/10.1186/s41235-021-00295-0

The original article (Muhl-Richardson et al., 2021) mistakenly omitted some Declarations. These Declarations have since been re-introduced.
