# Supplementary material for: Improved X-ray baggage screening sensitivity with ‘targetless’ search training
Source: Cogn Res Princ Implic. 2021 Apr 14;6:33. doi: 10.1186/s41235-021-00295-0 (PMC8046861; doi:10.1186/s41235-021-00295-0)
Supplement: Supplementary file 1 — Additional file 1. Supplementary Tables S1–S5. [file 41235_2021_295_MOESM1_ESM.docx]

| Table S1. Familiarisation Procedures and Stimuli | | | |
| --- | --- | --- | --- |
| **Experiment** | **Group** | **Familiarisation Procedure** | **Familiarisation Stimuli** |
| 1, 2, 3 | All groups | Viewing empty bags | 11 empty bags |
|  |  |  |  |
|  |  |  |  |
| 4 | ETST | Viewing empty bags | 11 empty bags |
|  |  |  |  |
|  |  |  |  |
|  | Practice Only | Viewing empty bags and pairs of objects from each category | 11 empty bags, 28 pairs of objects (one from each of the 14 target and 14 non-target categories) |

| Table S2. Training and Testing Stimulus Pool | | | | | | |  |
| --- | --- | --- | --- | --- | --- | --- | --- |
| **Pool of Possible Training and Testing Stimuli** | | | | | | |  |
| 52 empty bags (each in 4 orientations) | | | | | | |  |
| 416 bags (one silhouette shape; 50% target-present) | | | | | | |  |
|  | | | | | | |  |
| 196 threat items: ammunition (6), blunt weapons/axes (6), explosives (22), firearm magazines/components (22), large firearms (22), small firearms (21), grenades (6), knives/stabbing weapons (22), liquids/gases (22), throwing stars/knuckles (6), power tools (6), shrapnel (6), snips/scissors/pliers (22), trowels/wrenches (6) | | | | | | |  |
| 196 safe items: chargers/adapters (26), shoes (40), jewellery (12), cameras (12), kitchen items (10), computers (24), disc drives/media players (10), speakers (12), mobile telephones (12), hairdryers (6), glasses (8), irons (8), shavers/trimmers (6), purses/wallets (10) | | | | | | |  |
| 130 three-item bags, containing: chargers/adapters (52), shoes (80), jewellery (24), cameras (24), kitchen items (20), computers (46), disc drives/media players (20), speakers (24), mobile telephones (24), hairdryers (12), glasses (16), irons (16), shavers/trimmers (12), purses/wallets (20) | | | | | | |  |
| 152 practice bags (six objects; 50% target-present), containing: ammunition (2), blunt weapons/axes (1), explosives (9), firearm magazines/components (8), large firearms (13), small firearms (7), grenades (2), knives/stabbing weapons (6), liquids/gases (12), throwing stars/knuckles (2), power tools (1), shrapnel (2), snips/scissors/pliers (9), trowels/wrenches (2), chargers/adapters (69), shoes (75), jewellery (68), cameras (63), kitchen items (57), computers (69), disc drives/media players (49), speakers (75), mobile telephones (58), hairdryers (37), glasses (60), irons (52), shavers/trimmers (47), purses/wallets (57) | | | | | | |  |
| 208 bags (one object), containing: jewellery (6), stationery (16), chargers/adapters (12), bathroom items (14), toys (8), liquids/gases (6), clothes (8), shoes (8), firearm magazines/components (6), firearms (2), knives/blades (16), blunt weapons (2), batteries (2), speakers (6), ammunition (5), computers (12), cameras (8), explosives (13), mobile telephones (9), tools (6), food (7), grenades (1), kitchen items (8), shrapnel (2), tasers (1), miscellaneous (23), X-ray opaque items (1)  208 pairs of objects for 2AFC, including exactly one additional category-matching stimulus for each list above  168 bags (six objects; 50% target-present), containing: ammunition (3), blunt weapons/axes (4), explosives (9), firearm magazines/components (10), large firearms (5), small firearms (11), grenades (3), knives/stabbing weapons (11), liquids/gases (6), throwing stars/knuckles (3), power tools (4), shrapnel (3), snips/scissors/pliers (9), trowels/wrenches (3), chargers/adapters (65), shoes (80), jewellery (80), cameras (73), kitchen items (71), computers (81), disc drives/media players (75), speakers (69), mobile telephones (79), hairdryers (39), glasses (52), irons (52), shavers/trimmers (41), purses/wallets (67)  168 bags (six objects; 50% with additional spatial overlap; 50% target-present [balanced for overlap]), containing: ammunition (3 [2 overlap]), blunt weapons/axes (4 [1 overlap]), explosives (9 [1 overlap]), firearm magazines/components (10 [3 overlap]), large firearms (5 [2 overlap]), small firearms (11 [3 overlap]), grenades (3 [2 overlap]), knives/stabbing weapons (11 [1 overlap]), liquids/gases (6 [2 overlap]), throwing stars/knuckles (3 [2 overlap]), power tools (4 [2 overlap]), shrapnel (3 [2 overlap]), snips/scissors/pliers (9 [3 overlap]), trowels/wrenches (3 [2 overlap]), chargers/adapters (65), shoes (80), jewellery (80), cameras (73), kitchen items (71), computers (81), disc drives/media players (75), speakers (69), mobile telephones (79), hairdryers (39), glasses (52), irons (52), shavers/trimmers (41), purses/wallets (67) | | | | | | |  |
| Table S3. Training Stimuli by Experiment and Training Group | | | | |  |  |  |
| **Experiment** | **Group** | | **Training Stimuli** | |  |  |  |
|  |  | |  | |  |  |  |
| 1 | Conventional | | 196 threat items (all stimuli from pool) | |  |  |  |
|  |  | |  | |  |  |  |
|  | sTST | | 196 safe items (all stimuli from pool) | |  |  |  |
|  |  | |  | |  |  |  |
|  | CST | | 98 threat and 98 safe items: ammunition (3), blunt weapons/axes (3), explosives (11), firearm magazines/components (11), large firearms (11), small firearms (11), grenades (3), knives/stabbing weapons (11), liquids/gases (11), throwing stars/knuckles (3), power tools (3), shrapnel (3), snips/scissors/pliers (11), trowels/wrenches (3), chargers/adapters (13), shoes (20), jewellery (6), cameras (6), kitchen items (5), computers (12), disc drives/media players (5), speakers (6), mobile telephones (6), hairdryers (3), glasses (4), irons (4), shavers/trimmers (3), purses/wallets (5) | |  |  |  |
|  |  | |  | |  |  |  |
| 2, 3 | All Groups | | 196 safe items (all stimuli from pool) | |  |  |  |
|  |  | |  | |  |  |  |
| 4 | ETST | | 98 threat items (day one): ammunition (3), blunt weapons/axes (3), explosives (11), firearm magazines/components (11), large firearms (11), small firearms (11), grenades (3), knives/stabbing weapons (11), liquids/gases (11), throwing stars/knuckles (3), power tools (3), shrapnel (3), snips/scissors/pliers (11), trowels/wrenches (3) | |  |  |  |
|  |  | | 196 safe items (day one; all stimuli from pool) | |  |  |  |
|  |  | | 130 three-item bags (day one; all stimuli from pool) | |  |  |  |
|  |  | | 80 practice bags (day one; containing six objects; 50% target-present; randomly selected from pool) | |  |  |  |
|  |  | | 35 threat items (day two): ammunition (1), blunt weapons/axes (1), explosives (4), firearm magazines/components (4), large firearms (4), small firearms (4), grenades (1), knives/stabbing weapons (4), liquids/gases (4), throwing stars/knuckles (1), power tools (1), shrapnel (1), snips/scissors/pliers (4), trowels/wrenches (1) | |  |  |  |
|  |  | | 68 safe items (day two): chargers/adapters (9), shoes (14), jewellery (4), cameras (4), kitchen items (4), computers (5), disc drives/media players (4), speakers (4), mobile telephones (4), hairdryers (1), glasses (3), irons (3), shavers/trimmers (5), purses/wallets (4) | |  |  |  |
|  |  | | 45 three-item bags (day two; randomly selected from pool) | |  |  |  |
|  |  | | 28 practice bags (day two; containing six objects; 50% target-present; randomly selected from pool) | |  |  |  |
|  |  | |  | |  |  |  |
|  | Practice Only | | 152 practice bags (day one; containing six objects; 50 % target-present; all stimuli from pool) | |  |  |  |
|  |  | | 52 practice bags (day two; containing six objects; 50 % target-present; randomly selected from pool) | |  |  |  |
| Table S4. Testing Stimuli by Experiment | | | | | | | |
| **Experiment** | | | **Testing Stimuli** | | | | |
|  | | |  | | | | |
| 1, 2 | | | 168 bags (containing six objects; overall 50% target-present), containing: ammunition (3), blunt weapons/axes (4), explosives (9), firearm magazines/components (10), large firearms (5), small firearms (11), grenades (3), knives/stabbing weapons (11), liquids/gases (6), throwing stars/knuckles (3), power tools (4), shrapnel (3), snips/scissors/pliers (9), trowels/wrenches (3), chargers/adapters (65), shoes (80), jewellery (80), cameras (73), kitchen items (71), computers (81), disc drives/media players (75), speakers (69), mobile telephones (79), hairdryers (39), glasses (52), irons (52), shavers/trimmers (41), purses/wallets (67) | | | | |
| 3, 4 | | | 168 bags (containing six objects; overall 50% target-present; 50% with additional object overlap [equal present/absent split]), containing: ammunition (3 [2 overlap]), blunt weapons/axes (4 [1 overlap]), explosives (9 [1 overlap]), firearm magazines/components (10 [3 overlap]), large firearms (5 [2 overlap]), small firearms (11 [3 overlap]), grenades (3 [2 overlap]), knives/stabbing weapons (11 [1 overlap]), liquids/gases (6 [2 overlap]), throwing stars/knuckles (3 [2 overlap]), power tools (4 [2 overlap]), shrapnel (3 [2 overlap]), snips/scissors/pliers (9 [3 overlap]), trowels/wrenches (3 [2 overlap]), chargers/adapters (65), shoes (80), jewellery (80), cameras (73), kitchen items (71), computers (81), disc drives/media players (75), speakers (69), mobile telephones (79), hairdryers (39), glasses (52), irons (52), shavers/trimmers (41), purses/wallets (67) | | | | |
| Table S5. Experiment 1 Additional Two-way Mixed ANOVA Analysis Of *d’* For Test Phase Bags Containing No Items Repeated From Training (21 bags) Vs One Item Repeated From Training (53 bags) For Target-based Training And sTST Groups | | | | | | | |
| **Effect** | | | **ANOVA Result (*d’*)** | |  | | |
|  | | |  | | | | |
| Training Group  (target-based/sTST) | | | *F*(1,38) = 25.17, *p* < .001, η*_G_*^2^ = 0.29 | | | | |
| Stimulus Repetition  (zero/one) | | | *F*(1,38) = 0.25, *p* = .617 | | | | |
| Training Group x Stimulus Repetition | | | *F*(1,38) = 5.77, *p* = .021, η*_G_*^2^ = 0.05 | | | | |
| Follow-up *t*-tests | | | sTST group (*M* = 1.56, *SD* = 0.61) vs. target-based group (*M* = 0.56, *SD* = 0.36) for zero repeat bags, *t*(19) = 5.47, *p* < .001, *d* = 1.22.  sTST group (*M* = 1.34, *SD* = 0.61) vs. target-based group (*M* = 0.89, *SD* = 0.36) for one repeat bags, *t*(19) = 3.69, *p* = .002, *d* = 0.83. | | | | |
